# From prodigious volcanic degassing to caldera subsidence and quiescence at Ambrym (Vanuatu): the influence of regional tectonics

**DOI:** 10.1038/s41598-019-55141-7

**Published:** 2019-12-11

**Authors:** Tara Shreve, Raphaël Grandin, Marie Boichu, Esline Garaebiti, Yves Moussallam, Valérie Ballu, Francisco Delgado, Frédérique Leclerc, Martin Vallée, Nicolas Henriot, Sandrine Cevuard, Dan Tari, Pierre Lebellegard, Bernard Pelletier

**Affiliations:** 1 Université de Paris, Institut de physique du globe de Paris, CNRS, F-75005 Paris, France; 2 Univ. Lille, UMR 8518 - LOA - Laboratoire d’Optique Atmosphérique, F-59000 Lille, France; 3 CNRS, UMR 8518, F-59000 Lille, France; 4 Vanuatu Meteorology and Geohazards Department (VMGD), Port Vila, Vanuatu; 5 Laboratoire Magmas et Volcans (LMV), Université Clermont Auvergne, Clermont-Ferrand, 63170 France; 6 Lamont-Doherty Earth Observatory, Columbia University, New York, USA; 7 Laboratoire Littoral Environnement et Sociétés (LIENSs), Université de La Rochelle, La Rochelle, 17000 France; 8 Géoazur, Univ. Nice Sophia Antipolis (Univ. Côte d’Azur, CNRS, IRD, Observatoire de la Côte d’Azur), Géoazur UMR 7329, 250 rue Albert Einstein, Sophia Antipolis, 06560 Valbonne, France; 9 Géoazur, Institut de recherche pour le développement, Nouméa, 98800 New Caledonia

**Keywords:** Tectonics, Volcanology, Natural hazards

## Abstract

Eruptive activity shapes volcanic edifices. The formation of broad caldera depressions is often associated with major collapse events, emplacing conspicuous pyroclastic deposits. However, caldera subsidence may also proceed silently by magma withdrawal at depth, more difficult to detect. Ambrym, a basaltic volcanic island, hosts a 12-km wide caldera and several intensely-degassing lava lakes confined to intra-caldera cones. Using satellite remote sensing of deformation, gas emissions and thermal anomalies, combined with seismicity and ground observations, we show that in December 2018 an intra-caldera eruption at Ambrym preceded normal faulting with >2 m of associated uplift along the eastern rift zone and 2.5 m of caldera-wide subsidence. Deformation was caused by lateral migration of >0.4 cubic kilometers of magma into the rift zone, extinguishing the lava lakes, and feeding a submarine eruption in the rift edge. Recurring rifting episodes, favored by stress induced by the D’Entrecasteaux Ridge collision against the New Hebrides arc, lead to progressive subsidence of Ambrym’s caldera and concurrent draining of the lava lakes. Although counterintuitive, convergent margin systems can induce rift zone volcanism and subsequent caldera subsidence.

## Introduction

Broad caldera formation (>10 km in diameter) is often attributed to ignimbrite-forming, explosive eruptions^1^. For mafic to intermediate systems, however, caldera-forming processes may also be linked to the lateral propagation of dikes that arrest at depth^2–5^. Consequently, geological traces of associated magma discharge are often missing. Understanding caldera-forming processes thus relies heavily on contemporaneous observations, such as in 1968 at Fernandina and 2000 at Miyakejima^6,7^. Ambrym, a remarkably active volcanic island in the Vanuatu archipelago, hosts a 12-km wide caldera, making it one of the largest basaltic shield calderas on Earth^8,9^. Ambrym’s caldera has been previously interpreted as resulting from the collapse of a giant tuff cone resulting from a sequence of explosive phreatomagmatic eruptions^10^. Onset of caldera subsidence is dated around 2000 BP, based on two ^14^C dates of charcoal embedded in debris flows on the caldera rim and flank^11^. However, geological evidence for emplacement of voluminous ignimbrites coinciding with this dating is controversial^12–14^, raising questions about the process of caldera formation.

**Figure 1:**
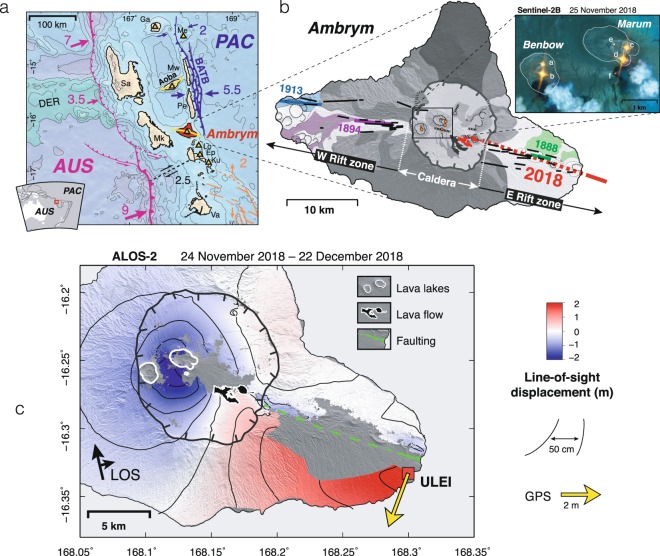
Tectonic and volcanic context of Ambrym. (**a**) Tectonic framework of the Central Vanuatu arc (modified after^71^). Collision of the D’Entrecasteaux Ridge (DER), carried by the Australian plate (AUS) leads to (1) along-strike variations of the convergence rate (pink arrows) of AUS w.r.t Pacific plate (PAC) across the New Hebrides subduction (pink) and (2) deformation of the Vanuatu arc on the border of the Pacific plate, accommodated by the back-arc thrust belt (BATB), giving rise to an uplifted bivergent thrust wedge. Arrows indicate local relative velocities across the BATB (blue) and Vate trough (orange). Velocities in cm/yr are from^72^. Yellow triangles are active volcanoes. Yellow lines indicate rift zones. Va: Vate; Ku: Kuwae; Ep: Epi; Lo: Lopevi; Mk: Malekula; Sa: Santo; Pe: Pentecost; Mw: Maewo; Ga: Gaua; Me: Mere Lava. (**b**) Simplified geological map of Ambrym (after^11^ and^18^). Younger volcanic formations are indicated by light shading. Locations of historical fissure vents along the rift zones are shown by thick colored lines. Approximate locations of the 2018 intra-caldera eruption and extra-caldera intrusion are indicated in red. Inset is a false-color Sentinel-2 image acquired on 25 November 2018 (R: band 12; G: band 11; B: band 08), showing six thermal anomalies associated with lava lakes and open vents. (**c**) Unwrapped ascending ALOS-2 interferogram spanning between 24 November 2018 and 22 December 2018. Blue (respectively red) indicates motion away from the satellite (respectively toward the satellite). Maps were generated with GMT version 5.4.3 (http://gmt.soest.hawaii.edu) and edited in Adobe Illustrator version 16.0.4 (https://www.adobe.com/products/illustrator.html).

Ambrym, in addition to its broad caldera, also hosts two well-defined straight rift zones oriented at N105°S, radiating bilaterally^10,11,15–17^ (Fig. 1). Ambrym’s caldera is the site of intense eruptive activity, with frequent strombolian eruptions originating from at least two permanent lava lakes within or on the flanks of the cones of Marum and Benbow as well as occasional intra-caldera lava flows, most recently in 1986, 1988 and 2015^12,18,19^. Continuous open-vent passive degassing from the lava lakes, first reported by Captain James Cook in 1774^20^, ranks Ambrym first in worldwide volcanic sulfur dioxide (SO_2_) emissions over the past decade^21–24^.

Between 1820 and 1937, 10 extra-caldera rift eruptions were reported at Ambrym^18^. Notably, in 1913, a phreatomagmatic eruption on the island’s west coast completely destroyed a large hospital^25^. Based on geochemical evidence, these eruptions are believed to be fed from a central reservoir situated beneath the caldera, and magma transported to the coast by lateral dikes^26^. However, the response of the caldera ring faults during these past lateral eruptions is undetermined, due to a lack of direct observations. Hence, whether the broad caldera of Ambrym should be interpreted as a relict structure, or should be considered an active fault system, remains an open question. More broadly, the relationship between the evolution of Ambrym’s caldera, the rift zone’s persistence, and the complex tectonic setting of the New Hebrides is yet to be explored.

## Results

### Precursory intra-caldera eruption

**Figure 2:**
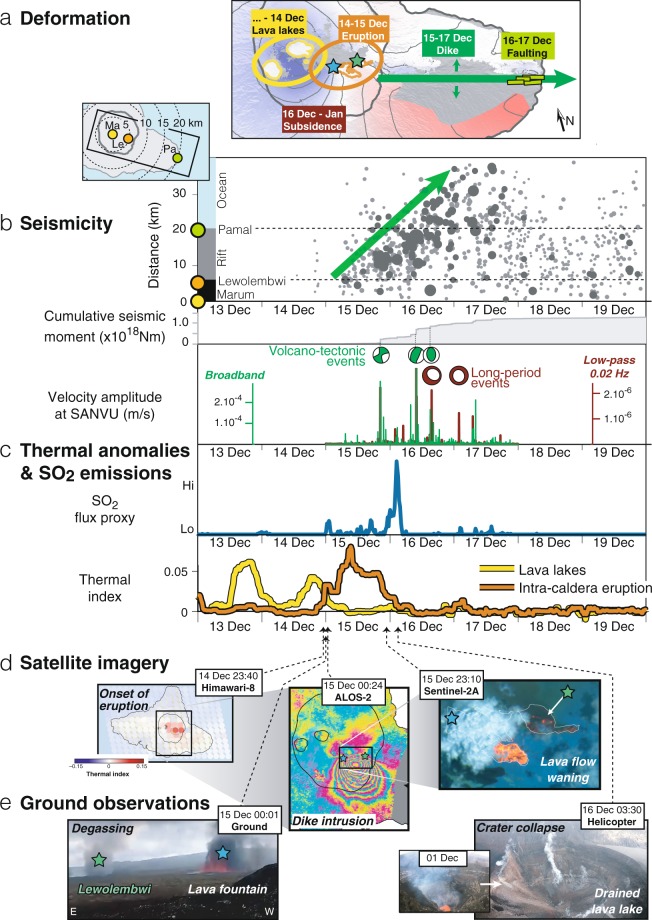
Temporal evolution of eruptive activity during the December 2018 event. (**a**) ALOS-2 interferogram of Fig. 1c (JAXA). (**b**) Upper panel: seismicity versus time, as a function of planimetric distance with respect to Marum lava lake. Symbol size increases with earthquake magnitude (dark grey: M > 3.5; light grey: M < 3.5). Green arrow highlights migration of seismicity along the east rift zone. Middle panel: cumulative seismic moment release. Focal mechanisms are from USGS (green) and GCMT (maroon). Lower panel: Absolute value of broadband (green) and low-passed (maroon) seismogram at SANVU Geoscope station (epicentral distance ~150 km)^31^. Spikes in the low-passed seismogram indicate detection of long period (LP) events. These events are not included in the cumulative seismic moment curve, which only includes volcano-tectonic (VT) events reported by VMGD. See Supplementary Fig. S14 for details and station location. (**c**) SO_2_ flux proxy (blue) and thermal index of the lava lakes (yellow) and intra-caldera eruption (orange) derived from Himawari-8. (**d**) Satellite images acquired during the course of the eruption. Left: Himawari-8 (multispectral, geostationary) (Japan Meteorological Agency). Center: ALOS-2 (ScanSAR) (JAXA). Right: Sentinel-2 (optical). (**e**) Ground observations. Left: gas emissions at Lewolembwi (green star) and lava fountaining associated with vent opening on the SE flank of Marum (blue star). Image courtesy of and copyright to John Tasso, Vanuatu Island Experience. Right: Comparison of lava lake crater at Benbow before and in the final hours of the eruption. Maps generated with GMT and edited in Adobe Illustrator.

On 14 December 2018, a volcano-seismic crisis begins at Ambrym when 8 M < 3 seismic events are detected inside the caldera between 13h00 and 20h00 UTC (Fig. 2b). Between 23h20 and 23h40 UTC, Himawari-8 geostationary satellite observations of thermal anomalies and SO_2_ emissions indicate the onset of an intra-caldera eruption (Fig. 2c,d, Supplementary Figs. S1, S2). Field observations (Fig. 2e) reveal that the eruption initiated along a N110° pre-existing fracture at 590 meters a.s.l. at Lewolembwi tuff ring (Supplementary Fig. S3), and was characterized by scoria deposits associated with lava fountaining. Petrological analysis of scoria indicates an erupted magma of basaltic-trachy-andesitic composition (Supplementary Table S4). Once the eruption begins, thermal anomalies associated with the lava lakes progressively disappear within 12 hours, suggesting a drop in lava lake level (Fig. 2c, Supplementary Fig. S1). A helicopter flight (on 16 December 03h30 UTC) confirms both drainage of all the lava lakes and the partial collapse of Benbow and Marum (Fig. 2e). A lava flow, accompanied by lava fountaining and producing SO_2_ and ash-rich emissions, is also emitted for ~24 hours from a second vent trending nearly N–S at 730 meters a.s.l. on the SE flank of Marum (Fig. 2c,e, Supplementary Fig. S3).

During this phase, surface deformation is measured with interferometric synthetic aperture radar (InSAR) thanks to the serendipitous acquisition of an image by the ALOS-2 satellite at 00h24 UTC on 15 December, about an hour after the eruption’s onset^27^ (Fig. 2d, Supplementary Table S1). The deformation field spanning 3 November to 15 December measures ~1.2 m of motion towards the satellite, consistent with an intra-caldera dike dipping 40°S and a maximum opening of ~2 m, yielding a total volume of intruded magma of ~34 × 10^6^ m^3^ (Supplementary Fig. S4). Until then, InSAR measures neither subsidence related to magma reservoir deflation, nor extra-caldera displacement, particularly no motion along the rift zones.

While Sentinel-2 satellite optical images indicate that lava flows reach their full extent on 15 December 23h10 (Fig. 2d), the eruption ends on 16 December around 07h00 UTC with a disappearance of thermal anomalies and an abrupt decrease of SO_2_/ash emissions (Fig. 2c). Spaceborne imaging of SO_2_ by Sentinel-5P TROPOMI sensor constrains the total mass of released SO_2_ during the eruption to at least ~50–60 kt (Supplementary Fig. S10), corresponding to a volume of degassed magma of ~13 × 10^6^ m^3^ (calculated assuming <5% crystal content and 0.075 wt% of sulphur in the melt^23^). First-order agreement with the volume of erupted material derived from mapping of the flow of ~10 × 10^6^ m^3^ (constrained by multiplying the spatial extent of ~1.95 × 10^6^ m^2^ for the lava flow, Supplementary Fig. S3, and an estimated average lava flow thickness of ~5 m extrapolated from 3D mapping of the previous 2015 intra-caldera lava flow, Supplementary Fig. S11) suggests that the magma remaining trapped in the dike did not contribute significantly to the observed degassing.

### Triggering of extra-caldera dike injection

**Figure 3:**
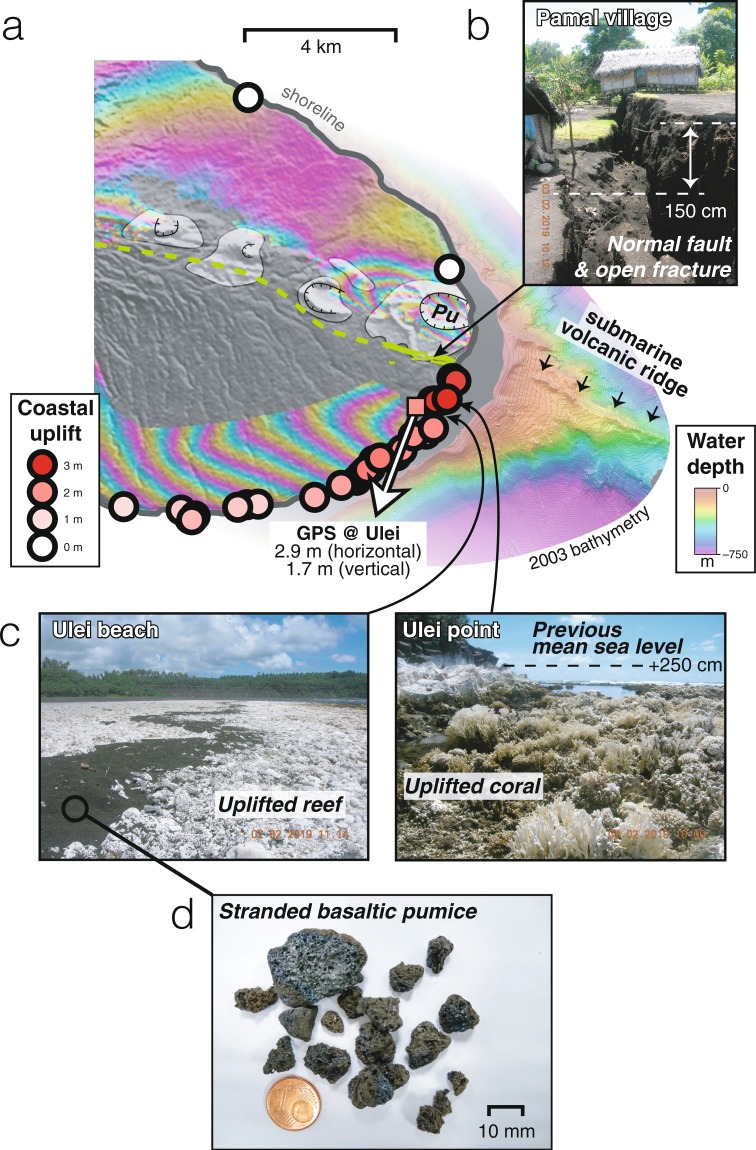
Faulting, coastal uplift and submarine eruption at the east coast of Ambrym. (**a**) Circles: coastal uplift derived from field mapping of dead coral and red algae. Green segments: fractures mapped in the field. Dashed green line: trace of the dike derived from SAR data. White arrow: motion of GPS Ulei site. Small black arrows indicate the location of a submarine volcanic ridge visible in high-resolution bathymetry acquired in 2003 (see Supplementary Methods). Pu: Pulvenu cone. Background is the wrapped ALOS-2 interferogram of Fig. 1c, where each fringe represents 12.1 cm of motion toward or away from the satellite. DEM ©DLR 2017. Maps generated with GMT and edited in Adobe Illustrator. (**b**) Normal faulting and fracturing at Pamal village. (**c**) Evidence for coastal uplift from dead coral. Black deposits consist of pumice stranded along the coast at Ulei. (**d**) Detail of a pumice sample.

**Figure 4:**
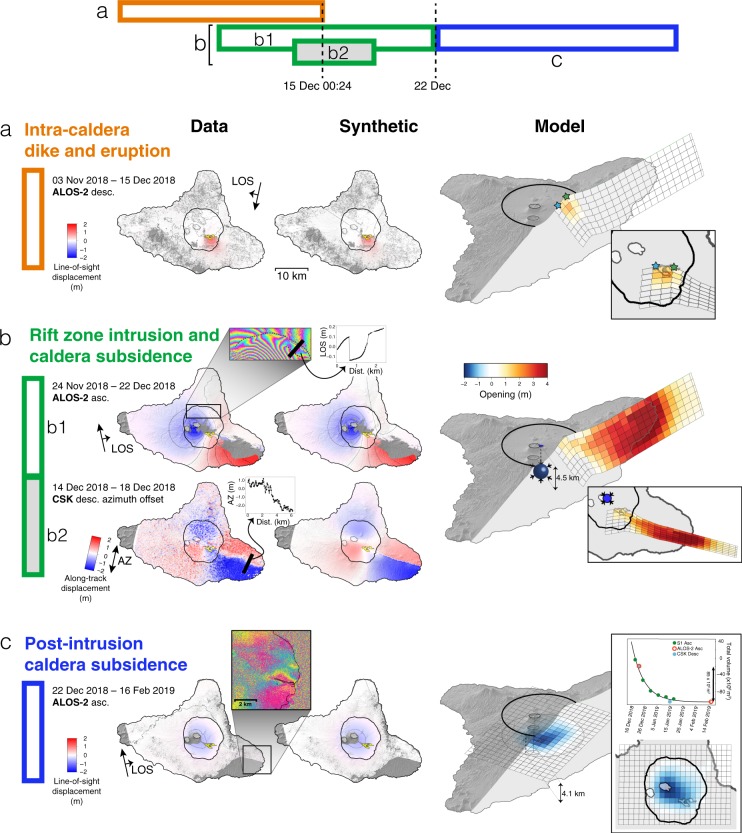
Deformation during and after the December 2018 event from InSAR. Left: data. Middle: synthetic. Right: model. (**a**) ALOS-2 interferogram covering the small-volume dike intrusion emplaced on 14 December (last image acquired on 15 December, 00h24). Dike inflation is indicated by red colors. Contraction or deflation is indicated by blue colors. (**b**) ALOS-2 interferogram (top) and COSMO-SkyMed azimuth offsets (bottom) covering the 15–18 December time interval. First inset shows local fringe discontinuities across the northern caldera rim, evidence of normal faulting. Second inset shows a profile of horizontal displacement across the dike. (**c**) ALOS-2 interferogram covering the post-intrusion caldera subsidence (first image acquired on 22 December). Inset to the left shows detail of deformation along the east coast. Inset to the right shows temporal evolution of post-intrusion subsidence derived from Sentinel-1, ALOS-2 and CSK (See Supplementary Fig. S7). Maps were generated with GMT version 5.4.3 (http://gmt.soest.hawaii.edu) and edited in Adobe Illustrator version 16.0.4 (https://www.adobe.com/products/illustrator.html).

Following a M_*w*_ 5.6 strike-slip earthquake on 15 December at 20h21, a sharp increase in the seismic moment release is detected, marking the beginning of magma propagation into the SE rift zone (Fig. 2b). A few hours before the intra-caldera eruption’s end, the lateral propagation of a voluminous dike is evidenced by a migration of seismicity from the caldera toward the eastern tip of the island, reaching 30 km from the eastern caldera rim by 17 December 12h00 UTC. Sub-pixel correlation of a Sentinel-2 optical image acquired on 15 December 23h10 UTC indicates that at that time the dike tip is leaving the caldera but did not reach farther than halfway to the east coast (Supplementary Fig. S15). On 16–17 December, local inhabitants report progressive fracturing at the coastal village of Pamal (13 km from the caldera border) (Fig. 3a,b). Joint inversion of SAR data (Supplementary Table S1) reveals ~3 m of opening along a >30 km long dike, extending from within the caldera to beyond the eastern coast^27^ (Figs. 2a and 4b). These SAR geodetic observations indicate the emplacement of a dike with a total volume of intruded magma between 419 and 532 × 10^6^ m^3^ (depending on the maximum depth and how far offshore the model extends, Supplementary Figs. S5, S6). Surface deformation across the trace of the dike is asymmetric, with more deformation to the south (Fig. 3a), indicating that the dike dips ~70° to the south. Due to this asymmetry, coastal uplift in excess of 2 meters occurred along the southeastern coast of the island, as later confirmed during a field campaign in early February and by a single GPS measurement at Ulei (Fig. 3a,c).

### Submarine eruption and caldera subsidence

The extremely narrow breadth of the faulted corridor observed above the dike at the surface, as small as 400 m along the east coast (Fig. 3b), indicates that the dike almost reached the surface. However, magma does not erupt from on-shore fractures and only minor gas emissions are detected from space until 17 December 14h00 UTC (Fig. 2c). The end of dike propagation, marked by an abrupt decrease of seismic moment release, takes place around 17 December 16h00 UTC (Fig. 2b). InSAR-derived models predict that maximum opening at the surface occurs offshore (Fig. 4b, Supplementary Fig. S6), suggesting a submarine eruption. This is confirmed on 18–19 December, when basaltic pumice is collected on the beach near Pamal and Ulei (Fig. 3c,d), indicating that lava erupted underwater. Although the depth and exact location of the underwater fissure are uncertain, the nature of erupted material (basaltic pumice) indicates a shallow (<100 m b.s.l) and high-rate underwater magma supply able to sustain a protective gas-rich envelope which allows pumice to cool before contact with sea water, preventing it from sinking^28^. This transport method differs from, for example, floating eruption products collected during basaltic eruptions in the Azores, when gas trapped in hollow cavities caused buoyant basaltic balloons to float to the ocean surface^29^. An alignment of volcanic cones visible in the bathymetry is consistent with an offshore prolongation of the rift zone, suggesting that similar submarine eruptions took place in the past (Fig. 3a).

In addition to the dike intrusion, we also measure >2 m of subsidence at Ambrym’s summit craters, consistent with deflation of a nearly symmetrical pressurized source, roughly centered on Marum at ~4.5 km depth, with a volume change ranging between 195 and 231 × 10^6^ m^3^ (depending on the depth and amount of post-eruptive deformation included in the data, Supplementary Fig. S7, Table S3) (Fig. 4c). The 2:1 volume ratio between the dike and deflating Mogi source is in fact consistent with mass conservation assuming standard magma compressibility and host-rock shear modulus^30^. Furthermore, SAR data indicate that caldera subsidence occurred between 15 December 00h24 and 18 December 06h10, hence was coeval to the extra-caldera dike intrusion (Fig. 4b2). There are several locations to the north of the caldera where the ascending ALOS-2 fringe pattern is discontinuous, indicating ~0.4 m slip along the caldera faults (Fig. 4b1, Supplementary Fig. S8). Two M_*w*_ ~5.6 earthquakes with vertical compensated-linear-vector-dipole (CLVD) focal mechanisms are recorded in the migration’s final 24 hours, on 16 December 15h34 UTC and 17 December 01h49 (Fig. 2b). These events are characterized by a long period (LP) content and long duration (exceeding 10 s of seconds) (Fig. 2b, Supplementary Fig. S14). At least 4 additional LP signals are recorded at station SANVU (~150 km away) on 16–17 December^31^. These LP events with CLVD mechanisms are consistent with caldera ring faulting or pressure drop within a reservoir^32^.

In the weeks following the dike emplacement, caldera-wide subsidence, reaching ~80 cm at Marum crater and decaying exponentially with a half-life of about 6 days, is measured using ALOS-2 and Sentinel-1 InSAR (Fig. 4c, Supplementary Fig. S7). Exponentially decaying subsidence is consistent with elastic response to magma outflow driven by pressure difference between the central reservoir and the eruption site^33^. Modeling of this subsidence indicates a horizontal sill at ~4.1 km depth deflating with a total volume change of ~85 × 10^6^ m^3^ (Fig. 4c). This sill-shaped post-intrusion deflation contrasts with the Mogi-shaped co-intrusion deflation, suggesting that the central reservoir consists of several storage levels^34^. Low-magnitude seismicity during this phase is shallow (<6 km depth) and located within the caldera, possibly related to continued caldera faulting visible in post-eruptive interferograms (Supplementary Figs. S12, S13).

Although no additional large-scale deformation is observed along the east rift zone after 22 December, a localized <12 cm discontinuity is measured across the fractures mapped along the SE coast (Fig. 4c), suggesting a continuation of the distal submarine eruption, driving the progressive drainage of the central magma reservoir, similar to the 2014 Bárarbunga or the 2018 Kilauea eruptions^35,36^. Field surveys confirm that the submarine eruption may have continued past the 27 December, as more pumice was observed on 3 February 2019 than on 27 December 2018. At the time of writing (August 2019), there has been no satellite detection of sulphur dioxide above background levels since late January 2019, contrasting starkly with the intense persistent degassing measured over the past decade^24^.

## Discussion

**Figure 5:**
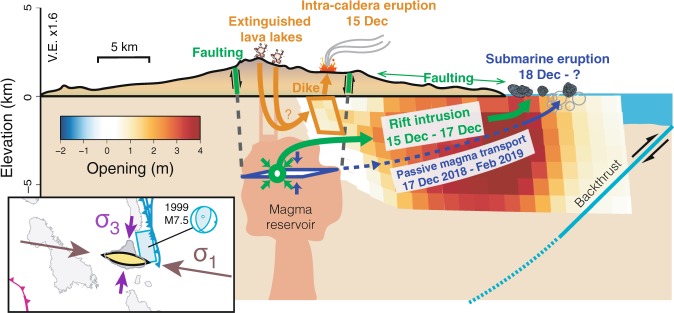
Conceptual model of the December 2018 event. Magma injection into the rift zone occurred in two stages. First, on 14 December, a small-volume dike was emplaced in the eastern part of the caldera. The dike breached the surface and fed an eruption lasting ~24 hours. Deflation of Ambrym’s magma plumbing system as a result of the intra-caldera eruption induced a drop of pressure in the magma column, leading to lava lake drainage. On 15–16–17 December, a large-volume dike injection took place along the east rift zone, likely fed through the conduit opened by the previous intra-caldera dike. During its propagation and inflation, the voluminous dike intrusion induced intense faulting and fissuring above its upper edge, especially at the coastal village of Pamal, suggesting a shallow depth of emplacement. However, the dike did not produce any onshore eruption nor any substantial degassing. From 18 December, pumice washed up on the eastern shore of Ambrym indicating a submarine eruption. Continued subsidence of Ambrym caldera during the weeks following this eruption, as well as continuation of localized faulting at Pamal (Fig. 3c), suggest a prolongation of the submarine eruption without substantial additional inflation of the main dike, compatible with passive magma transport from Ambrym’s central reservoir toward the submarine eruption site. The inset is a zoom on Ambrym’s tectonic setting, emphasizing that Ambrym’s rift zone orientation is sub-parallel to the regional maximum compressive stress, allowing us to interpret Ambrym as a large tension fracture. The activated plane and focal mechanism of the 1999 thrust earthquake are noted on the sketch in light blue.

The December 2018 Ambrym diking event sheds light on the stress state that prevails at the scale of Ambrym island, while providing insight into the magma storage conditions beneath Ambrym’s caldera (Fig. 5). Joint analysis of remote sensing and seismicity demonstrates that the condition initiating the rift intrusion in December 2018 was the creation of an open fracture at Lewolembwi, connecting the caldera’s localized magma supply to the rift zone. Once such a connection is made, a blade-like fluid-filled crack (dike) is able to travel tens of kilometers away from its source, neither erupting at the surface, nor degassing significantly to the atmosphere. To sustain lateral magma propagation, pressure in the dike (P_*m*_) must be greater than the host rock’s minimum principal stress normal to the dike plane (*σ*_3_, positive), a difference defined as the driving stress (P_*d*_) (P_*d*_ = P_*m*_ − *σ*_3_)^37–39^. Furthermore, to travel horizontally for long distances, there must be a strong horizontal gradient of driving stress in the direction of magma propagation^40^. Magmatic systems in an extensionally-loaded host rock (low *σ*_3_) do not necessarily require high magma overpressures to drive large diking events (high P_*d*_)^41^. When comparing diking events at Icelandic and Hawaiian volcanoes, dike widths tend to be thicker in Iceland (higher P_*d*_ in Iceland), but dikes reach the surface more often in Hawaii (higher P_*m*_ in Hawaii). The >3 m dike thickness at Ambrym brings us to the conclusion that P_*d*_ is high without having a large P_*m*_^41^. A similar conclusion was drawn after the dike intrusions and lava lake drainage of the 2002 Nyiragongo flank eruption^42,43^. In spite of enhanced thermal activity and increased lava lake vigor (Supplementary Figs. S16, S17), an absence of uplift in the months to years prior to the 2018 Ambrym crisis is evidenced (Supplementary Fig. S9), consistent with this inferred lack of overpressure^44^. The fact that the central magma reservoir was able to store a large volume of magma (0.4 km^3^) and sustain dike propagation over large distances without prior overpressurization is consistent with vigorous supply of a relatively volatile-poor magma^23^.

The thick 2018 dike–similar in width to dikes at plate boundaries in Iceland or Afar^45^–and energetic earthquake migration indicate that lithospheric stresses primed for magma injection drove lateral magma propagation. Assuming a minimum volume of intruded magma in the 2018 eruption (400 × 10^6^ m^3^), we calculate a minimum expansion rate along Ambrym’s rift of ~2 cm/yr (assumptions: last event = 81 years ago; SE rift length = 25 km; height = 10 km). Gravitationally-induced extension can be plausibly discarded as a mechanism generating these stresses, due to the lack of curvature of Ambrym’s rift zone^46^ and to the fact that the 2018 rift zone intrusion led to uplift of the entire south-eastern part of the island, hence working against gravity. On the other hand, tectonic stresses induced by the proximity of regional faults involved in the convergence between the Pacific and Australian plates may provide the stress conditions driving rift development at Ambrym. Ambrym is facing the collision-subduction of the D’Entrecasteaux Ridge (DER) and, farther to the north, of the West Torres Massif (WTM) (Fig. 1a). The DER and WTM perturb the Vanuatu arc, resulting in the late Quaternary uplift of the western Santo-Malekula islands^47,48^ and the growth of a back-arc thrust belt (BATB), uplifting the eastern islands of Pentecost-Maewo (Fig. 1a)^49–51^. Ambrym’s east coast is located at the southern tip of the BATB, which was last activated in the thrusting 1999 M_*w*_ 7.5 earthquake^52,53^. We note that the focal mechanisms during the 2018 diking event have P-axis orientations consistent with this regional compression (Fig. 2b). This triggered seismicity does not appear consistent with stress change caused by the dike inflation (increased compressive stress oriented perpendicular to the dike, i.e. N20°) but rather reflects the regional stress state (*σ*_3_ oriented N110°), revealing the dominant overprint of this background compressive stress^54^. In this context, Ambrym may be envisioned as a giant tension fracture oriented sub-parallel to the local maximum compression axis *σ*_1_ (inset of Fig. 5). The importance of the regional stress field’s maximum compression axis was also emphasised after the 2000 lateral dike intrusion at Miyakejima, likewise situated in a convergent margin setting^55^.

The 2018 diking event illustrates how tectonically-induced stresses drive magma transport into Ambrym’s well-defined rift zone, efficiently siphoning magma away from the caldera in a relatively silent manner for an observer at the surface. Similar to past events in hot-spot basaltic volcanoes of the Galapagos, especially in 1968 at Fernandina^56,57^, caldera subsidence was associated with non-explosive activity, thereby leaving little geological trace at the surface. Rather, the elevated rate of Ambrym’s volcanic activity witnessed in the past decades contrasts with the near-complete muting of degassing and thermal activities at the surface during and since the December 2018 extra-caldera dike intrusion (Supplementary Fig. S17). Modulation of volcanic activity at Ambrym’s lava lakes over historical times^18^ may therefore be reinterpreted as resulting from recurrent pumping of magma into the rift zone, leading to episodic subsidence of the caldera floor. Open-vent degassing in the years prior to the 2018 eruption may have allowed for an increasing availability of non-overpressurized magma, with lava lakes acting as piezometers of a magma plumbing system that is well-connected to the surface. This offers a glimpse into a system where magma ascent, lateral magma transport, and caldera formation are controlled by regional compressive tectonics.

## Methods

### Himawari processing

Himawari-8 is a meteorological satellite operated by the Japan Meteorological Agency, providing multispectral observations from a geostationary orbit at 140.7°E. The Advanced Himawari Imager (AHI) covers 16 channels spanning visible to thermal infrared, and acquires images every 20 minutes. Images have a resolution of ~2.3 km at the location of Ambrym.

Following^58^, a raw thermal index is calculated by computing the normalized difference between top-of-atmosphere brightness temperatures from infrared channels centered on 10.41 microns and 3.9 microns:

1 $$T{I}^{raw}=\frac{B{T}_{3.9\mu m}-B{T}_{10.4\mu m}}{B{T}_{3.9\mu m}+B{T}_{10.4\mu m}}.$$

In order to mitigate the impact of clouds and diurnal variations in brightness temperature, a background thermal index is estimated by extracting the raw thermal index for a reference pixel situated at the border of the caldera, that is not affected by the thermal anomaly of lava and ash emissions while sharing similar ground properties as the intra-caldera pixels. This background thermal index is subtracted from the raw thermal index, yielding a corrected thermal index.

The thermal index time series of Fig. 2c is then estimated by calculating the thermal index averaged in two regions corresponding to the lava lakes and lava flows. Supplementary Fig. S1 shows the same time series of Fig. 2c on a longer time window.

The SO_2_ flux proxy is estimated in two steps. First, following^59^, a SO_2_ column amount proxy is calculated for each pixel by differencing top-of-atmosphere brightness temperatures from infrared channels centered on 10.41 microns and 8.5 microns:

2 $$C{A}_{S{O}_{2}}\propto B{T}_{10.4\mu m}-B{T}_{8.5\mu m}$$

Then, the time series of SO_2_ flux proxy ${Q}_{S{O}_{2}}$ in Fig. 2c is calculated by summing, for each acquisition, the value of $C{A}_{S{O}_{2}}$ for all pixels with a $C{A}_{S{O}_{2}}$ greater than 4 K within a ~50 × 35 km box centered on Ambrym (dashed polygon in Supplementary Fig. S2b). The threshold is intended to distinguish the signal associated with the presence of volcanic SO_2_ from background oscillations. Supplementary Fig. S2a shows the SO_2_ flux proxy of Fig. 3c on a longer time window, plotted both on a linear scale and a logarithmic scale.

### InSAR processing

The SAR images used in this study are listed in Supplementary Table S1. Processing of SAR data from the SLC level to wrapped, unfiltered interferograms is performed using the Interferometric SAR scientific computing environment (ISCE)^60^ for ALOS-2 StripMap (SM3) and Cosmo-SkyMed (CSK) StripMap data and Generic Mapping Tool’s software GMTSAR^61^ for ALOS-2 wideswath (WD1) data, with additional post-processing using the NSBAS^62^. Topographic fringes are removed using DLR’s TanDEM-X 12 meter Global (TDX) DEM (an average of DEMs acquired between 14 January 2011 and 22 November 2014)^63^. Interferograms are filtered with a weighted power spectrum filter^64^, followed by a cascading high-pass filter, especially useful in the areas with a high-gradient fringe rate and on the vegetated flanks^65^. An iterative, coherence-based unwrapping method is then used^65^, which we will call MPD, and is a module in NSBAS (see Supplementary Methods). The final unwrapped interferograms are then geocoded.

CSK interferograms have low coherence across the island, due to vegetation, atmospheric effects, and a high rate of deformation. Therefore, CSK descending pixel offsets, which complement the ascending ALOS-2 measurements, were exploited to measure deformation during the rift zone intrusion and the post-intrusion caldera subsidence. After unwrapping and geocoding, swaths F1 and F2 are merged for ALOS-2 WD1 interferograms. We also merge along-track frames for ALOS-2 SM3 interferograms, to ensure that the far-field signal (Pentecost island to the north and Lopevi, Paama, and Namuka islands to the south) is included in the inversion.

### Geodetic modeling

#### Inversion procedure

We then perform the same inversion procedure for each of the three datasets, respectively corresponding to the (a) intra-caldera dike, (b) rift zone intrusion and caldera subsidence, and (c) post-intrusion caldera subsidence (Fig. 4). To focus on the first-order geometry of the pressure sources, we mask localized, near-field signals that may bias the model misfits (i.e. masking localized deformation within 2 km of the dike trace, deformation close to the craters that may be due to conduit pressurization, or subsidence due to lava flow cooling and compaction).

After downsampling the data using a distance-based averaging, we then run a non-linear inversion to find the first order geometry of the pressure sources^66^ (See Supplementary Table S2). The forward model includes dislocations^67^ and Mogi sources^68^. We find that the intra-caldera phase (a) is best explained by a single inflating dike, whereas the rift zone intrusion and caldera subsidence in phase (b) require an inflating dike and a deflating Mogi. We find that post-intrusion caldera subsidence in phase (c) cannot be explained by the same deflating Mogi as in phase (b), whereas a laterally extensive sill-like deflating source better reproduces the deformation measured in post-eruptive interferograms.

Following this non-linear inversion, the geometry of the pressurized sources is held fixed, and we then performed a constrained least squares inversion to investigate separately the distributed opening of the intra-caldera dike, extra-caldera rift intrusion (modeling the residual deformation field after removing the synthetic deformation from the deflating Mogi), and the closing of the post-intrusion sill^45,69^ (see Supplementary Methods).

During the rift zone intrusion, there was significant deformation offshore, limiting the model’s resolution. Therefore, we perturb the geometry of the dike used to model the rift zone intrusion by extending the depth, as well as the length offshore, by several kilometers. We derive four end-member models to determine a reasonable range of volumes, taking into account uncertainties imposed by the lack of model resolution (Supplementary Fig. S6). Final volumes range from 419 to 532 × 10^6^ m^3^, with an “average” model chosen with a reasonable misfit, which extends 6 km offshore and 6 km along depth.

Once the distributed opening along the rift-zone dike is determined, we perform a final iteration to ensure that the initial removal of the Mogi model’s synthetic deformation does not propagate significant model errors into the distributed opening inversion. We subtract the synthetic deformation field derived from the “average” model from the original datasets, and rerun the nonlinear inversion to solve for the Mogi depth and volume change. The volume and depth do not change significantly (<6% and <3%, respectively).

#### Temporal evolution of post-intrusion subsidence

In addition to an ALOS-2 interferogram and CSK pixel offsets measuring deformation after the rift zone intrusion (Fig. 4c), ascending Track 81 Sentinel-1 images were also acquired every 6 days, starting from 19 December 2018. The magnitude of deformation measured in the 6-day pairs decreases with time, allowing us to investigate the temporal evolution of the subsidence. The full extent of the deformation signal is not captured in some of the interferograms because coherence is limited to within the unvegetated part of the caldera. Conversely, ALOS-2 interferograms exhibit a better coherence, which allows for mapping the deformation outside the caldera. However, the temporal resolution of ALOS-2 data is insufficient to capture the temporal evolution of this transient post-intrusion subsidence signal.

In order to combine the high temporal resolution of Sentinel-1 and the high coherence of ALOS-2, we design a specific strategy by (a) first estimating the parameters (shape, depth) of the deflating source from the deformation pattern visible in ALOS-2 data and (b) solving for the temporal evolution of the deflation source by fitting the deformation signal projected in the line of sight (LOS) against the deformation measured in Sentinel-1 data.

To exploit the high coherence of the ALOS-2 interferogram, we use the same inversion procedure as mentioned above to invert for closing on a horizontal sill, fixed to a depth of 4.1 km, with patches 1 km ×  1 km (Supplementary Fig. S4). The depth was derived from an initial non-linear inversion of a closing Okada plane. We then project the synthetic deformation field into the ascending Sentinel-1 LOS, and scale the model to fit the 15 interferograms spanning 19 December 2018 to 18 January 2019. The scaling is determined such that:

3 $${\gamma }_{k}=\frac{{\bar{u}}_{k}}{{\bar{u}}_{model}},$$

where *k* is the interferogram index, ${\bar{u}}_{k}$ and ${\bar{u}}_{model}$ are the average velocities of the data and model, respectively (i.e. the displacement divided by the time span). Here, *γ*_*k*_ is the scalar for interferogram *k* representing the rate of deformation in the time interval spanned by the interferogram. See Supplementary Methods for more details.

We then multiply each scalar by the total magma volume loss in the initial inversion (−85 × 10^6^ m^3^), and perform a least squares inversion to find volume loss for each acquisition (Fig. 4c)^70^. An exponential is fit to the volume loss vs. time,

4 $$F(t)=A\cdot \exp (\frac{-t}{B})-C,$$

such that *A* = 0.144 m^3^, *B* = 9.55 days, *C* = 0.1 m^3^, and *t* is the time is days since 16 December 2018, which corresponds with the inferred time of onset of caldera subsidence based on seismicity. The half-life of the exponential decay is thus ~6.6 days.

#### Removing post-intrusion subsidence

The datasets spanning the rift zone intrusion and main caldera subsidence also span the beginning of the post-intrusion subsidence. We scale the post-intrusion synthetic deformation field, using the exponential described above, to remove post-intrusion deformation from the co-intrusion ALOS-2 and CSK data. However, after rerunning the non-linear inversion on these corrected interferograms, the RMS was not improved–24.03 (without removal) vs. 24.74 (with removal). This may be because the source geometry does not remain constant throughout the entire post-intrusion subsidence phase (especially in the days immediately after the intrusive event, 18–22 December, which are not covered by the initial post-intrusion ALOS-2/CSK inversion). We therefore decide to proceed without removing the post-intrusion deformation.

## Supplementary information

Supplementary information

## References

[1] Druitt TH, Sparks RSJ (1984). On the formation of calderas during ignimbrite eruptions. Nature.

[2] Walker GPL (1993). Basaltic-volcano systems. Geol. Soc. London, Special Publ..

[3] Gudmundsson A (1998). Formation and development of normal-fault calderas and the initiation of large explosive eruptions. Bull. Volcanol..

[4] Acocella V (2007). Understanding caldera structure and development: An overview of analogue models compared to natural calderas. Earth-Science Rev..

[5] Michon L, Massin F, Famin V, Ferrazzini V, Roult G (2011). Basaltic calderas: Collapse dynamics, edifice deformation, and variations of magma withdrawal. J. Geophys. Res. Solid Earth.

[6] Simkin T, Howard KA (1970). Caldera collapse in the Galápagos Islands, 1968. Science.

[7] Geshi N, Shimano T, Chiba T, Nakada S (2002). Caldera collapse during the 2000 eruption of Miyakejima Volcano, Japan. Bull. Volcanol..

[8] Pike RJ (1978). Volcanoes on the inner planets: Some preliminary comparisons of gross topography. Proc. Lunar Planet Sci. Conf..

[9] Wood CA (1984). Calderas: a planetary perspective. J. Geophys. Res..

[10] Robin C, Eissen JP, Monzier M (1993). Giant tuff cone and 12-km-wide associated caldera at Ambrym Volcano (Vanuatu, New Hebrides Arc). J. Volcanol. Geotherm. Res..

[11] Mccall, G. J. H., Lemaitre, R. W., Malahoff, A., Robinson, G. P. & Stephenson, P. J. The Geology and Geophysics of the Ambrym Caldera, New Hebrides. In Symposium Volcanoes and their Roots, 681–696 (Oxford, England, 1969).

[12] Németh K, Cronin SJ (2008). Volcanic craters, pit craters and high-level magma-feeding systems of a mafic island-arc volcano: Ambrym, Vanuatu, South Pacific. Geol. Soc. London, Special Publ..

[13] Németh K, Cronin SJ, Stewart RB, Charley D (2009). Intra- and extra-caldera volcaniclastic facies and geomorphic characteristics of a frequently active mafic island-arc volcano, Ambrym Island, Vanuatu. Sedimentary Geol..

[14] Cronin, S. J. & Németh, K. Where are the giant tuff cone and ignimbrites of Ambrym? A more conventional story of mafic volcanism at Ambrym Volcano, Vanuatu. In *Geological Society of New Zealand 50th Annual Conference*, 21–22 (Geological Society of New Zealand, Kaikoura, New Zealand, 2005).

[15] Stephenson, P. *et al*. Geology of Pentecost and Ambrym, 1:100.000. *New Hebrides Geological Survey Sheet* **6**. (1976).

[16] MacFarlane, A., Carney, J. N., Crawford, A. J. & Greene, H. Vanuatu - A review of the onshore geology. In H.G., G. & F.L., W. (eds.) *Geology and offshore resources of Pacific island arcs - Vanuatu region, Circum-Pacific, no. 8 in Earth Science Series*, 45–91 (Circum-Pacific Council for Energy and Mineral Resources, 1988).

[17] Picard C, Monzier M, Eissen J-P, Robin C (1994). Concomitant evolution of tectonic environment and magma geochemistry, Ambrym volcano (Vanuatu, New Hebrides arc). Geol. Soc. London, Special Publ..

[18] Eissen, J. P., Blot, C. & Louat, R. Chronology of the historic volcanic activity of the New Hebrides island arc from 1595 to 1991. *Tech. Rep., ORSTOM, Nouméa* (1991).

[19] Coppola D, Laiolo M, Cigolini C (2016). Fifteen years of thermal activity at Vanuatu’s volcanoes (2000–2015) revealed by MIROVA. J. Volcanol. Geotherm. Res..

[20] Gregory J (1917). The Ambrym eruptions of 1913–1914. Geol. Mag..

[21] Bani P (2009). Surge in sulphur and halogen degassing from Ambrym volcano, Vanuatu. Bull. Volcanol..

[22] Bani P (2012). First estimate of volcanic SO_2_ budget for Vanuatu island arc. J. Volcanol. Geotherm. Res..

[23] Allard P (2015). Prodigious emission rates and magma degassing budget of major, trace and radioactive volatile species from Ambrym basaltic volcano, Vanuatu island Arc. J. Volcanol. Geotherm. Res..

[24] Carn SA, Fioletov VE, Mclinden CA, Li C, Krotkov NA (2017). A decade of global volcanic SO2 emissions measured from space. Sci. Reports.

[25] Németh K, Cronin SJ (2011). Drivers of explosivity and elevated hazard in basaltic fissure eruptions: The 1913 eruption of Ambrym Volcano, Vanuatu (SW-Pacific). J. Volcanol. Geotherm. Res..

[26] Firth C, Handley H, Turner S, Cronin S, Smith I (2016). Variable conditions of magma storage and differentiation with links to eruption style at Ambrym volcano, Vanuatu. J. Petrol..

[27] Hamling, I. J., Cevuard, S. & Garaebiti, E. Large scale drainage of a complex magmatic system: Observations from the 2018 eruption of Ambrym volcano, Vanuatu. *Geophys. Res. Lett*. (2019).

[28] Newton, A. *Ocean-transported pumice in the North Atlantic*. Ph.D. thesis, University of Edinburgh (2000).

[29] Kueppers U, Nichols AR, Zanon V, Potuzak M, Pacheco JM (2012). Lava balloons-peculiar products of basaltic submarine eruptions. Bull. Volcanol..

[30] Rivalta E, Segall P (2008). Magma compressibility and the missing source for some dike intrusions. Geophys. Res. Lett..

[31] Institut de Physique du Globe de Paris and Ecole et Observatoire des Sciences de la Terre de Strasbourg (EOST), GEOSCOPE - French Global Network of broadband seismic stations, 10.18715/GEOSCOPE.G (1982).

[32] Shuler A, Ekström G, Nettles M (2013). Physical mechanisms for vertical-CLVD earthquakes at active volcanoes. J. Geophys. Res. Solid Earth.

[33] Dvorak JJ, Okamura AT (1987). A hydraulic model to explain variations in summit tilt rate at Kilauea and Mauna Loa volcanoes. US Geol. Surv. Prof. Pap.

[34] Baker S, Amelung F (2012). Top-down inflation and deflation at the summit of Kilauea Volcano, Hawai’i observed with InSAR. J. Geophys. Res..

[35] Neal CA (2018). The 2018 rift eruption and summit collapse of Kilauea Volcano. Science.

[36] Gudmundsson MT (2016). Gradual caldera collapse at Bárdarbunga volcano, Iceland, regulated by lateral magma outflow. Science.

[37] Buck WR, Einarsson P, Brandsdóttir B (2006). Tectonic stress and magma chamber size as controls on dike propagation: Constraints from the 1975–1984 Krafla rifting episode. J. Geophys. Res. Solid Earth.

[38] Pollard DD, Delaney PT, Duffield WA, Endo ET, Okamura AT (1983). Surface deformation in volcanic rift zones. Tectonophysics.

[39] Rubin, A. M. & Pollard, D. D. Origin of blade-like dikes in volcanic rift zones. In Decker, R., Wright, T. & Stauffer, P. (eds.) Volcanism in Hawaii Professional Paper, 1449–1470, 1350 edn (U.S. Geol. Surv., 1987).

[40] Grandin R (2012). Thickness control of lateral dyke intrusion at mid-ocean ridges. Earth Planet. Sci. Lett..

[41] Rubin AM (1990). A comparison of rift-zone tectonics in Iceland and Hawaii. Bull. Volcanol..

[42] Komorowski J-C (2004). The January 2002 flank eruption of Nyiragongo volcano (DRC): chronology, evidence for a tectonic rift trigger, and impact of lava flows on the city of goma. Acta Vulcanologica.

[43] Wauthier, C., Cayol, V., Kervyn, F. & d’Oreye, N. Magma sources involved in the 2002 Nyiragongo eruption, as inferred from an InSAR analysis. *J. Geophys. Res. Solid Earth* 117 (2012).

[44] Cervelli P (2002). The 12 September 1999 Upper East Rift Zone dike intrusion at Kilauea Volcano, Hawaii. J. Geophys. Res. Solid Earth.

[45] Grandin, R. *et al*. September 2005 Manda hararo-dabbahu rifting event, Afar (Ethiopia): Constraints provided by geodetic data. *J. Geophys. Res. Solid Earth* **114**, 10.1029/2008JB005843 (2009).

[46] Nakamura K (1977). Volcanoes as possible indicators of tectonic stress orientation - principle and proposal. J. Volcanol. Geotherm. Res..

[47] Taylor FW, Isacks BL, Jouannic C, Bloom AL, Dubois J (1980). Coseismic and Quaternary vertical tectonic movements, Santo and Malekula Islands, New Hebrides Island Arc. J. Geophys. Res..

[48] Collot J, Daniel J, Burne R (1985). Recent tectonics associated with the subduction/collision of the d’entrecasteaux zone in the central new hebrides. Tectonophysics.

[49] Louat R, Pelletier B (1989). Seismotectonics and present-day relative plate motions in the Tonga-Lau and Kermadec-Havre region. Tectonophysics.

[50] Pelletier B, Meschede M, Chabernaud T, Roperch P, Zhao X (1994). Tectonics of the Central New Hebrides Arc, North Aoba Basin. Proceedings of the Ocean Drilling Program, 134 Scientific Results.

[51] Taylor FW (1995). Geodetic measurements of convergence at the New Hebrides island arc indicate arc fragmentation caused by an impinging aseismic ridge. Geology.

[52] Lagabrielle Y, Pelletier B, Cabioch G, Regnier M, Calmant S (2003). Coseismic and long-term vertical displacement due to back arc shortening, central Vanuatu: Offshore and onshore data following the Mw 7.5, 26 November 1999 Ambrym earthquake. J. Geophys. Res..

[53] Regnier M, Calmant S, Pelletier B, Lagabrielle Y, Cabioch G (2003). The Mw 7.5 1999 Ambrym earthquake, Vanuatu: A back arc intraplate thrust event. Tectonics.

[54] Roman DC, Heron P (2007). Effect of regional tectonic setting on local fault response to episodes of volcanic activity. Geophys. Res. Lett..

[55] Ueda H (2005). Magma intrusion and discharge process at the initial stage of the 2000 activity of Miyakejima, Central Japan, inferred from tilt and GPS data. Geophys. J. Int..

[56] Bagnardi M, Amelung F (2012). Space-geodetic evidence for multiple magma reservoirs and subvolcanic lateral intrusions at Fernandina Volcano, Galápagos Islands. J. Geophys. Res. B: Solid Earth.

[57] Munro DC, Rowland SK (1996). Caldera morphology in the western Galápagos and implications for volcano eruptive behavior and mechanisms of caldera formation. J. Volcanol. Geotherm. Res..

[58] Wright R, Flynn L, Garbeil H, Harris A, Pilger E (2002). Automated volcanic eruption detection using MODIS. Remote. sensing environment.

[59] Prata, A. & Kerkmann, J. Simultaneous retrieval of volcanic ash and SO2 using MSG-SEVIRI measurements. *Geophys. Res. Lett*. **34** (2007).

[60] Rosen, P. A., Gurrola, E., Sacco, G. F. & Zebker, H. The InSAR scientific computing environment. In *EUSAR 2012; 9*^*th*^*European Conference on Synthetic Aperture Radar*, 730–733 (2012).

[61] Sandwell D, Mellors R, Tong X, Wei M, Wessel P (2011). Open radar interferometry software for mapping surface deformation. Eos, Transactions Am. Geophys. Union.

[62] Doin, M.-P. *et al*. Presentation of the small baseline NSBAS processing chain on a case example: the Etna deformation monitoring from 2003 to 2010 using Envisat data. In *Proceedings of the ESA’Fringe 2011 Workshop’, Frascati, Italy*, (19–23 September 2011), 19–23 (2011).

[63] Moore I. D., Grayson R. B., Ladson A. R. (1991). Digital terrain modelling: A review of hydrological, geomorphological, and biological applications. Hydrological Processes.

[64] Rosen PA, Hensley S, Peltzer G, Simons M (2004). Updated Repeat Orbit Interferometry Package Released. Eos.

[65] Grandin R., Doin M.-P., Bollinger L., Pinel-Puyssegur B., Ducret G., Jolivet R., Sapkota S. N. (2012). Long-term growth of the Himalaya inferred from interseismic InSAR measurement. Geology.

[66] Tarantola A, Valette B (1982). Generalized nonlinear inverse problems solved using the least squares criterione. Rev. Geophys..

[67] Okada Y (1985). Surface Deformation Due to Shear and Tensile Faults in a Half-Space. Bull. Seismol. Soc. Am..

[68] Mogi K (1958). Relations between the eruptions of various volcanoes and the deformations of the ground surfaces around them. Bull. Earthuake Res. Inst..

[69] Jolivet R., Simons M., Agram P. S., Duputel Z., Shen Z.-K. (2015). Aseismic slip and seismogenic coupling along the central San Andreas Fault. Geophysical Research Letters.

[70] Berardino P, Fornaro G, Lanari R, Sansosti E (2002). A new algorithm for surface deformation monitoring based on small baseline differential SAR interferograms. IEEE Transactions on Geosci. Remote. Sens..

[71] Pelletier B, Calmant S, Pillet R (1998). Current tectonics of the Tonga-New Hebrides region. Earth Planet. Sci. Lett..

[72] Bergeot, N. *et al*. Horizontal and vertical interseismic velocity fields in the Vanuatu subduction zone from GPS measurements: Evidence for a central Vanuatu locked zone. *J. Geophys. Res. Solid Earth* **114** (2009).

