# Supplementary material for: From prodigious volcanic degassing to caldera subsidence and quiescence at Ambrym (Vanuatu): the influence of regional tectonics
Source: Sci Rep. 2019 Dec 11;9:18868. doi: 10.1038/s41598-019-55141-7 (PMC6906323; doi:10.1038/s41598-019-55141-7)
Supplement: Supplementary file 1 — Supplementary information [file 41598_2019_55141_MOESM1_ESM.pdf]

From prodigious volcanic degassing to caldera subsidence and quiescence at Ambrym (Vanuatu): the influence of regional tectonics- Supplementary Information

Tara Shreve[a,*], Raphaël Grandin[a,*], Marie Boichu[b,c], Esline Garaebiti[d], Yves Moussallam[e,f], Valérie Ballu[g], Francisco Delgado[a], Frédérique Leclerc[h], Martin Vallée[a], Nicolas Henriot[b], Sandrine Cevuard[d], Dan Tari[d], Pierre Lebellegard[i], and Bernard Pelletier[i]

[a]Université de Paris, Institut de physique du globe de Paris, CNRS, F-75005 Paris, France
[b]Univ. Lille, UMR 8518 - LOA - Laboratoire d'Optique Atmosphérique, F-59000 Lille, France
[c]CNRS, UMR 8518, F-59000 Lille, France
[d]Vanuatu Meteorology and Geohazards Department (VMGD), Port Vila, Vanuatu
[e]Laboratoire Magmas et Volcans (LMV), Université Clermont Auvergne, 63170, France
[f]Lamont-Doherty Earth Observatory, Columbia University, New York, USA
[g]Laboratoire Littoral Environnement et Sociétés (LIENSs), Université de La Rochelle, 17000, France
[h]Géoazur, Univ. Nice Sophia Antipolis (Univ. Côte d'Azur, CNRS, IRD, Observatoire de la Côte d'Azur), Géoazur UMR 7329, 250 rue Albert Einstein, Sophia Antipolis 06560 Valbonne, France.
[i]Géoazur, Institut de recherche pour le développement, Nouméa, 98800, Nouvelle-Calédonie
[*]shreve@ipgp.fr; grandin@ipgp.fr

Supplementary Methods

InSAR processing

MPD uses the interferogram's coherence, calculated in the previous filtering step, to optimize the unwrapping path. It begins unwrapping the phase at a seed pixel that is above a coherence threshold. MPD will slightly decrease the coherence threshold at each iteration, and will continue the phase unwrapping with nearby pixels that have a coherence above this new threshold. Pixels with coherence beneath a minimum threshold will not be unwrapped. Bridges defined manually are used to connect fringes that are determined to be continuous, but which pass through incoherent regions. Unwrapping errors that could not be corrected with bridges are shifted manually by adding integer multiples of 2π. Each pixel in the unwrapped interferogram has an iteration number, which represents a proxy for coherence and phase reliability. This value is used to mask the interferograms after the unwrapping is completed.

Geodetic Modeling

Inversion procedure

The Classic Slip Inversion (CSI) software is used to setup the forward problem[1]. Before performing the least squares inversion, the synthetic deformation from the Mogi source is removed from interferograms spanning the rift zone intrusion and caldera subsidence, and the synthetic deformation from the non-linear inversion is used to shift the phase values of the spatially disconnected islands by an appropriate integer multiple of 2π.

Datasets are downsampled by a distance-based averaging, with a denser spacing in the region of high gradients in the deformation field, and coarser spacing in regions with less deformation (i.e. in the far-field)[1]. We define a starting and minimum block size ($S_{block,start}$ and $S_{block,min}$), a characteristic distance D_{char}, and an exponent α (≥ 1), such that the block is divided into four smaller blocks if the following condition is met:

$$D_{block} - D_{char} < {S_{block}}^{\alpha}, \tag{1}$$

where D_{block} is the current distance from the source and S_{block} is the current block size. After downsampling, the size of the decimated data vector is between 200 and 1000 points per image.

The data covariance matrix for each interferogram is created by calculating the covariogram from 5000 random samples in a region where no deformation is measured, after removing a phase ramp. The covariogram, γ_h, is calculated from data binned every 0.5 km for InSAR measurements and every 0.2 km for pixel offsets, and is fit by an exponential of the form $\gamma_h = \gamma_0 - (\sigma_d^2) \cdot \exp(\frac{-x}{\lambda_d})$, where x is the distance between two points. This allows for empirically estimating the values of γ_0, σ_d and λ_d. The covariance matrix, $C_d(i,j)$, is populated according to:

$$C_d(i,j) = \sigma_d^2 \exp(-\frac{D}{\lambda_d}), \tag{2}$$

where D is the distance between the elements in the matrix.

We assume the same dike geometry for both the intra-caldera dike and the extra-caldera rift intrusion, because the ALOS-2 ascending and CSK descending datasets span the emplacement of both the intra-caldera and extra-caldera dikes. Within the caldera, the westernmost portion of the dike has a dip of 40°, and becomes progressively more vertical to the east, with a final dip of 70° along the rift zone intrusion (See Fig. 4). The patches are 1.7 km wide along-dip and 1.17 km long along-strike.

The model covariance matrix controls the roughness of the model, and is defined as[2]:

$$C_m(i,j) = \frac{\sigma_m \lambda_0}{\lambda_m}^2 \exp(\frac{-||i,j||_2}{\lambda_m}), \tag{3}$$

where i and j are two distinct patches, σ_m is the correlation amplitude, λ_0 is a normalizing distance, λ_m is the correlation length, and $||i,j||_2$ is the distance between patches i and j . We fix λ_0 to 4 km, and in order to find optimal values for the correlation length λ_m and amplitude σ_m, we plot an L-curve of maximum slip vs. misfit for various values of λ_m and σ_m (Fig. S5). The constrained least squares inversion utilizes a Sequential Least Squares Programming algorithm from the python package SciPy to invert for opening on each of the patches. We constrain the opening along the dike to the positive, and we do not allow for slip. A planar ramp is also fit and removed from the data.

Temporal evolution of post-intrusion subsidence

We determine $\bar{u}_k$ and $\bar{u}_{model}$ by finding the average velocities within a 30x30 pixel box in the southern portion of the caldera (168.144324/168.168324/-16.284603/-16.260603). After exploring several different locations inside and outside the caldera, the scalar calculated in this location explains the most data variance. Several Sentinel-1 interferograms and their residuals ($u_{res} = u_k - (u_{model} * \gamma_k)$) are shown in Fig. S7. All final scalars are shown in Tab. S3.

GPS data

The GPS displacement vector in ULEI is obtained by comparison of a series of measurements conducted before (15–16 July 2018) and after (04–09 February 2019) the volcanic event. The ground marker is sealed in a concrete basement and has been installed in 1999 following the 26 November 1999 M_w7.5 Ambrym earthquake. This point belongs to the Vanuatu geodetic network and is remeasured periodically as part of a long-term post-seismic deformation study. Data have been collected using a dual frequency Topcon GB1000 receiver and a Topcon PG_A1 with ground-plane antenna installed on a mast of known height above the ground marker. We calculated daily positions in the ITRF2014 reference frame with a IPPP strategy (Precise Point Positioning with Integer ambiguity fixing) using the processing software GINS developed at CNES/GRGS[3], with GRG orbits and clocks products and IGS igs14.atx file for antenna calibration. Wet tropospheric delays are estimated every two hours based on VMF1 global mapping functions[4] and GPT2 empirical slant delay model GPT2[5].

Bathymetric data

Bathymetric data were acquired from 8–22 December 2003 during the Terralis cruise (PI: B. Pelletier) on board R/V Alis (IRD), using the multibeam echo sounder (MBES) SIMRAD EM1002 (Kongsberg). This MBES is designed to acquire high-resolution data of coastal areas down to a depth of 1000 m, operating with a frequency of 93 kHz. Acoustic signal duration is shorter than 2 ms, inducing a vertical accuracy of the seafloor measurements $\leq$8 cm. The MBES consists of 111 beams ($2^\circ \times 2^\circ$ beamwidth) that are distributed over an angular coverage of 150° across track, as to acquire seafloor measurements equidistant transversally (every 6 or 20 m for a seafloor at 100 of 300 mbsl respectively). Data were acquired at a vessel speed <8 knots inducing an along-track inter-ping distance of a few meters at 100 m water-depth and $\sim$ 9 m at 1000 m water-depth. The GPS system was however able to provide position with an absolute horizontal accuracy of only 10–15 m.

Bathymetric data were processed using the CARAIBES and SONARSCOPE softwares (©IFREMER). The processing consisted mainly in the correction of the navigation when artefacts were present, and in the automatic and manual filtering of the bathymetric data. These data were merged into a Digital Elevation Model gridded at 25 m/pixel in a WGS84 geographic reference system. They are represented in Figure 3a.

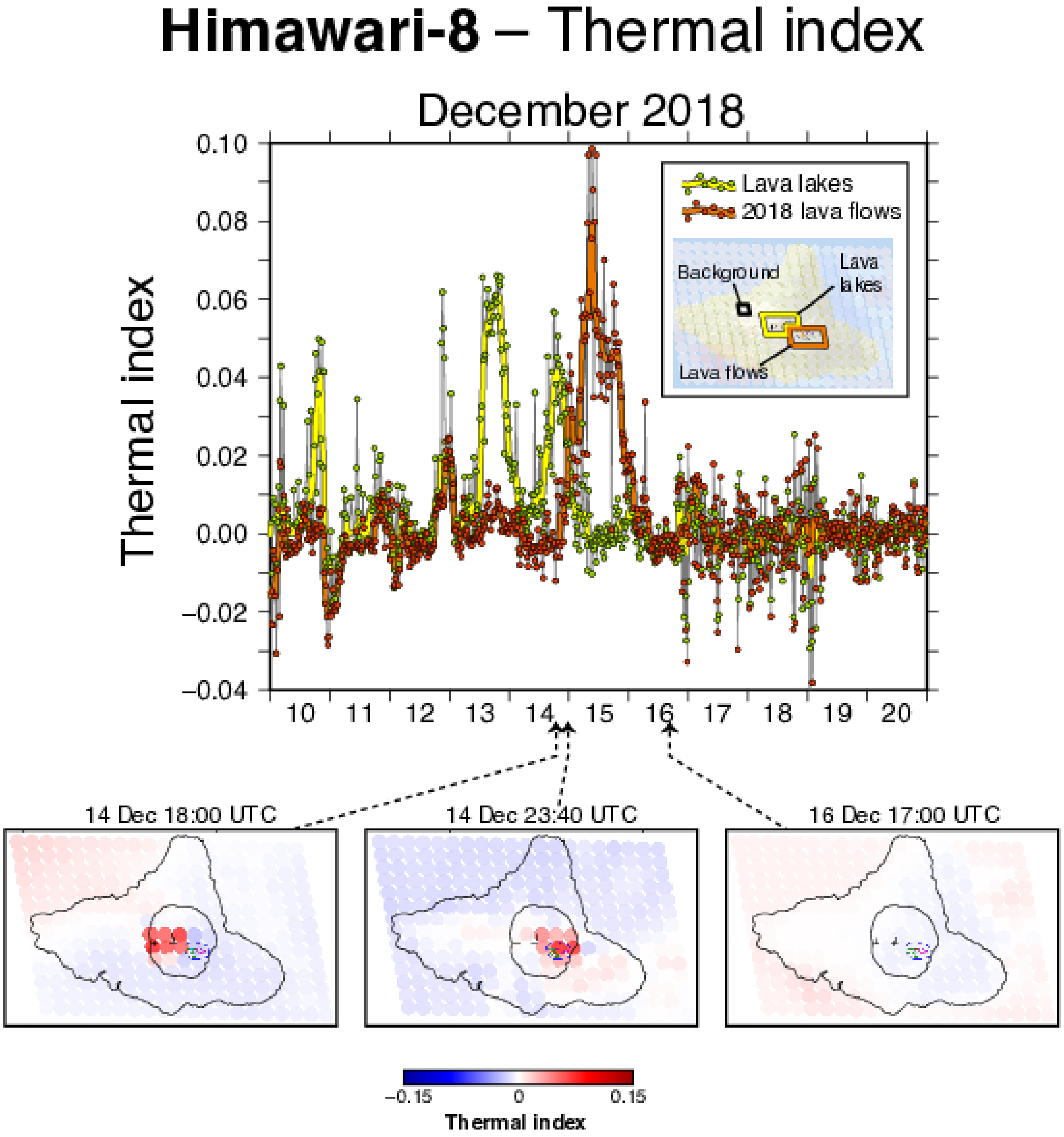

Figure S1. Time series of thermal index at Ambrym from geostationary Himawari-8 imagery. Upper panel: time series of the normalized thermal index[6] retrieved for Himawari-8 pixels corresponding to the lava lakes (yellow) and the 2018 intra-caldera lava flows (orange). Map in the inset shows the pixels used for the definition of these two locations. Color circles represent the thermal index with respect to a reference background pixel (shown in black in inset) derived from individual images acquired every 20 minutes. Thick color lines represent the smoothed time series after applying a median filter over 10 points (i.e. filter width: 200 minutes). Lower panel: maps of normalized thermal index at three different times corresponding to (a) lava lake activity prior to the 2018 eruption, (b) onset of the eruption and (c) lack of thermal anomaly after the end of the eruption. Himawari-8 imagery provided by AERIS/ICARE Data and Service Center and Japan Meteorological Agency. Maps were generated with GMT version 5.4.3 (http://gmt.soest.hawaii.edu) and edited in Adobe Illustrator version 16.0.4 (https://www.adobe.com/products/illustrator.html).

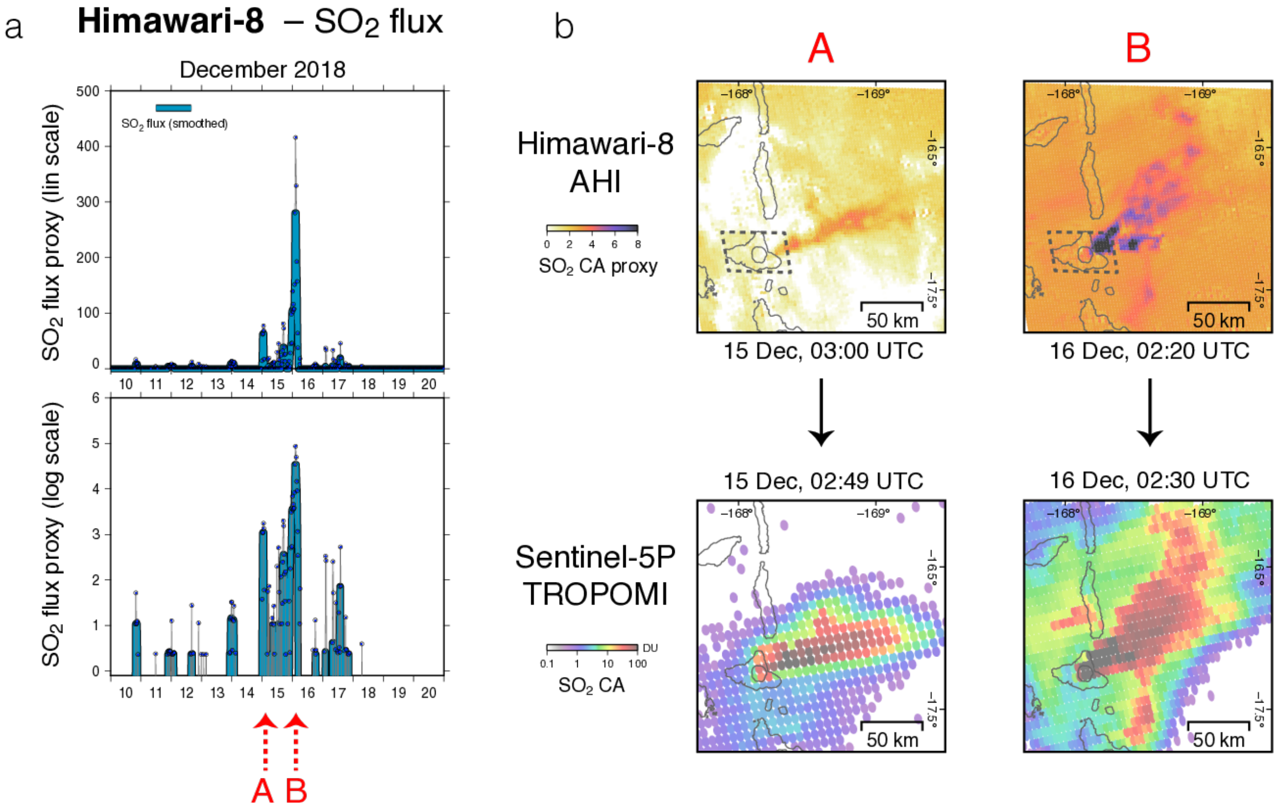

Figure S2. **a**. Upper panel: time series of SO_2 flux proxy at Ambrym from geostationary Himawari-8 imagery. Blue dots show the SO_2 flux proxy calculated from each acquisition, at 20 minutes interval. Thick blue curve is the filtered time series. Y-axis scaling is linear. Lower panel: same as upper panel, with a logarithmic scaling on the Y-axis. **b**. Comparison of SO_2 maps derived from Himawari-8 (this study) and Sentinel-5P TROPOMI acquired at approximately the same time. A: 15 December 2018, 03h00 UTC (Himawari-8), 02h49 (TROPOMI). B: 16 December, 02h20 UTC (Himawari-8), 02h30 (TROPOMI). Grey dashed box shows the area used for calculating the SO_2 flux proxy. Himawari-8 imagery provided by AERIS/ICARE Data and Service Center and Japan Meteorological Agency.

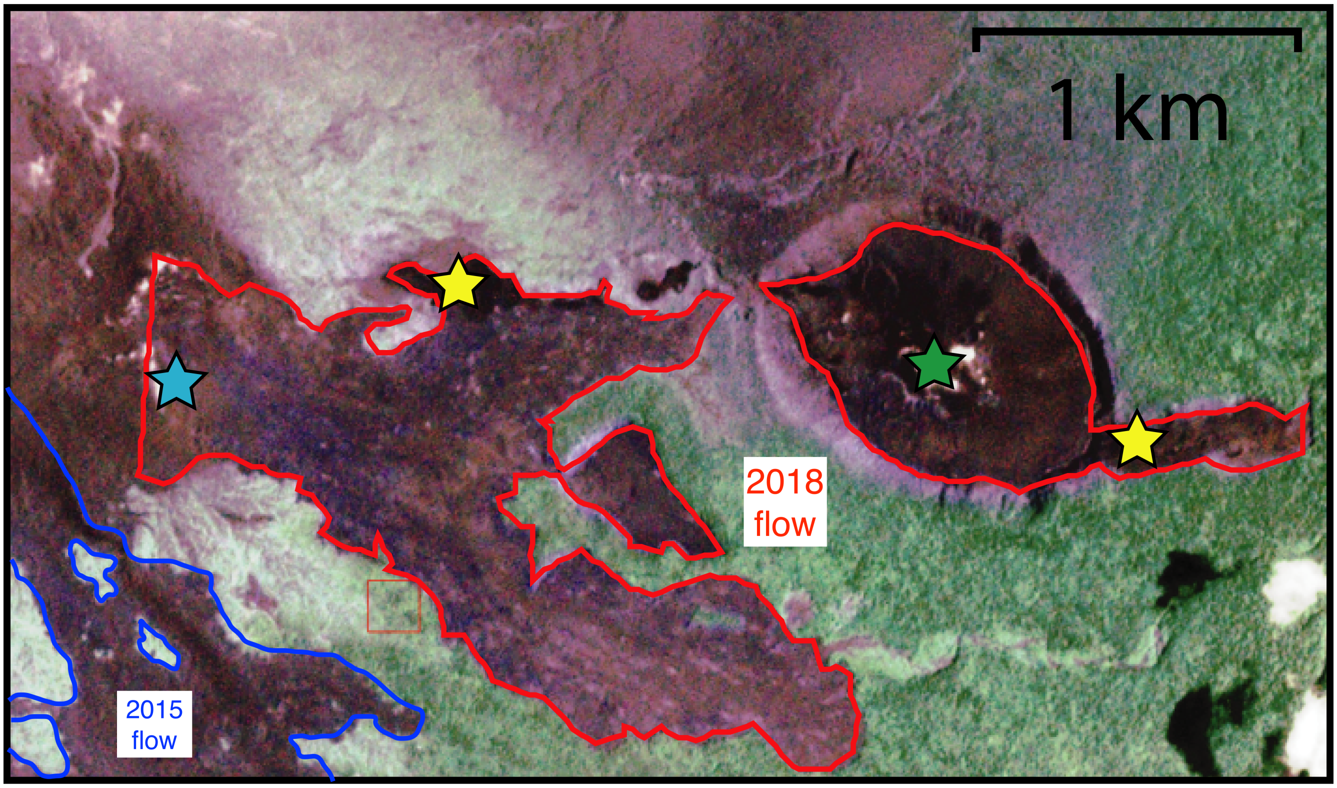

Figure S3. **Planet Labs optical image, acquired on January 31, 2019.** The full extent of the intra-caldera lava flow is outlined in red. The blue and green stars correspond, respectively, to the sources of lava fountaining and degassing visible in Fig. 2e in the text. Yellow stars mark the location of thermal anomalies noticed in the Sentinel-2 image acquired at 23h10 on 15 December (Fig. 2d), possibly indicating the secondary vents. Optical imagery obtained from Planet Labs (`https://api.planet.com`)[7]. Map created with ENVI Classic version 5.5.1 (https://www.harris.com/solution/envi).

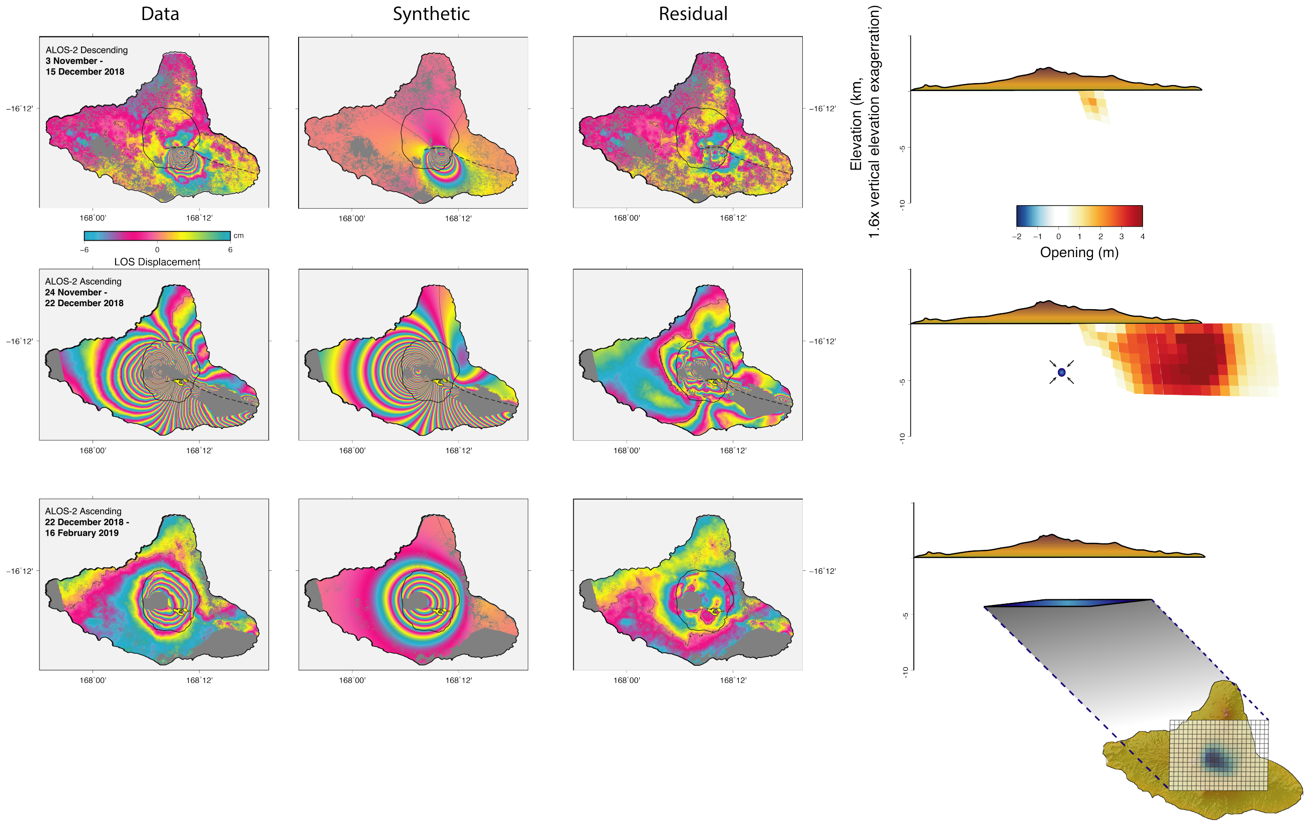

Figure S4. Wrapped data, model, and residuals. The first three columns correspond, respectively, to the data, model, and residual of interferograms displayed in Fig. 4 in the text, wrapped such that one fringe = 12.12 cm (ALOS-2 fringe rate). For all three rows, the model is derived from a distributed opening constrained least square's inversion, as outlined in the supplementary text. The opening distribution is shown in the fourth column. First row: data, model, and residual for a single interferogram used to invert for opening of the initial, intra-caldera dike intrusion feeding the effusive eruptions. We fix the dike geometry (See Fig. 4 in text) to be the same for both this inversion and the rift zone intrusion. Second row: data, model, and residual for the most coherent of the four datasets used to invert for the rift zone intrusion and caldera subsidence (see Table S1). Remaining residuals may be related to oversimplified geometry of the decompressing source (Mogi, fixed at a depth of 4.5 km), as well as the fact that the near field (<2 km) signal along the dike was masked, and therefore not fit by the inversion. Third row: data, model, and residual for one of three datasets (see Table S1) used to invert for exponentially decaying subsidence lasting more than 2 months after the rift zone intrusion. The horizontal sill is fixed at a depth of 4.1 km.

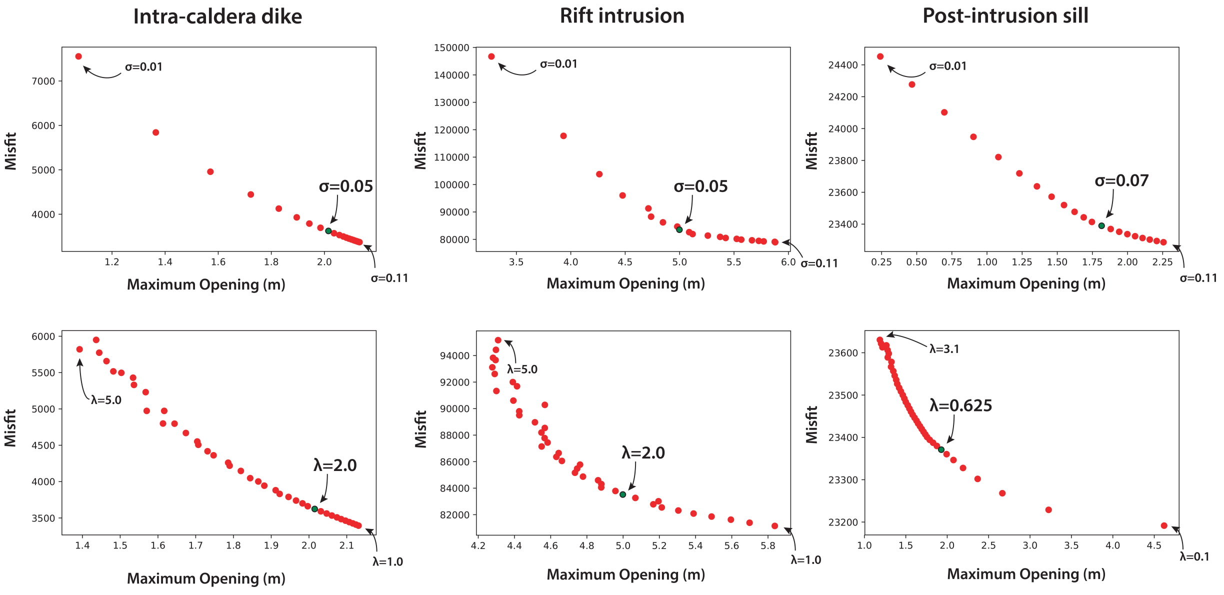

Figure S5. L-curve analysis for model smoothing parameters. To find the optimal amplitude of correlation (σ) and correlation distance (λ) for the model smoothing parameters, we iterate through a range of σ and λ values, and plot the model misfit vs. maximum opening. The maximum opening acts as a proxy for the model roughness, with a larger opening corresponding to a rougher model (smaller λ or larger σ). We initially run the iteration of σ with a fixed value of λ (2 for the dike intrusions, 0.8 for the sill), and then subsequently iterate for λ given the optimal values for σ. To remain consistent between the intra-caldera dike and rift zone intrusion, we choose the same σ and λ for both of these inversions.

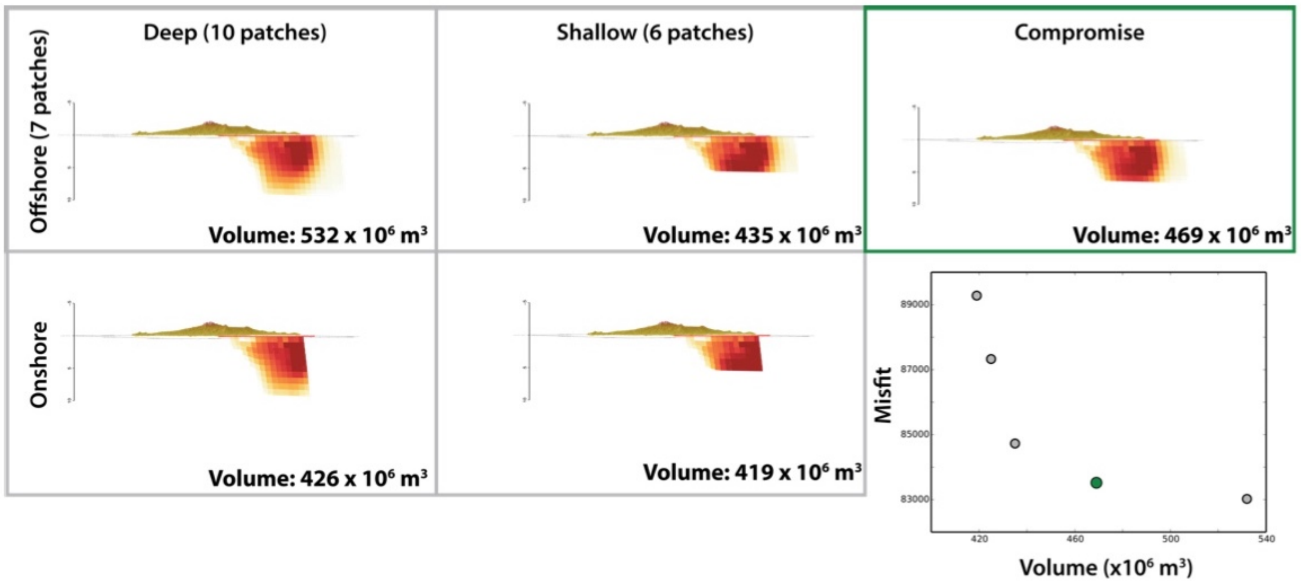

Figure S6. End-member distributed opening models. Four end-member models, resulting from the constrained least square's inversion of distribution opening, showing the uncertainty of volume estimates, ranging from 419 to 532×10^6 m^3. Total volume depends on the final model depth and extent offshore. Based on the misfit of these end-members, a compromise is found with the dike extending offshore ~6 km and to a depth of ~6 km.

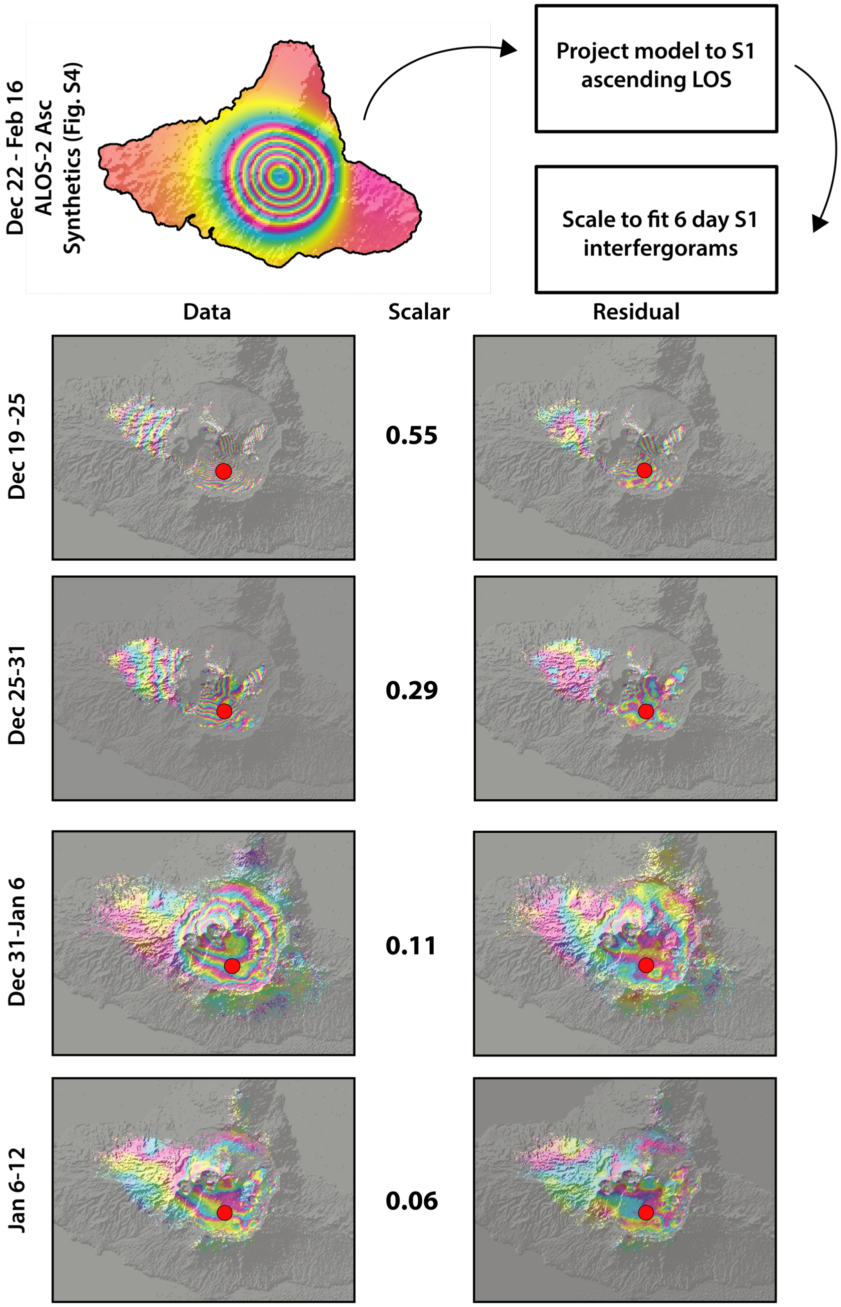

Figure S7. Temporal evolution of post-intrusion subsidence from Sentinel-1 interferograms. Synthetic deformation of a sill model, derived from the inversion of CSK pixel offsets and an ALOS-2 interferogram (22 Dec 2018 – 16 Feb 2019) that is coherent across the entire island, is scaled to fit the displacement field in Sentinel-1 6-day interferometric pairs, in order to investigate the temporal evolution of post-intrusion caldera subsidence. The lack of coherence in the S1 interferograms precluded a standard time series inversion. Instead, a scaling constant is determined for all 15 interferograms spanning 19 Dec – 18 Jan 2019 (assuming the source geometry has not changed), and the sill model volume (85×10^6 m^3) is scaled accordingly for each interferogram. Refer to the supplementary text for more detail.

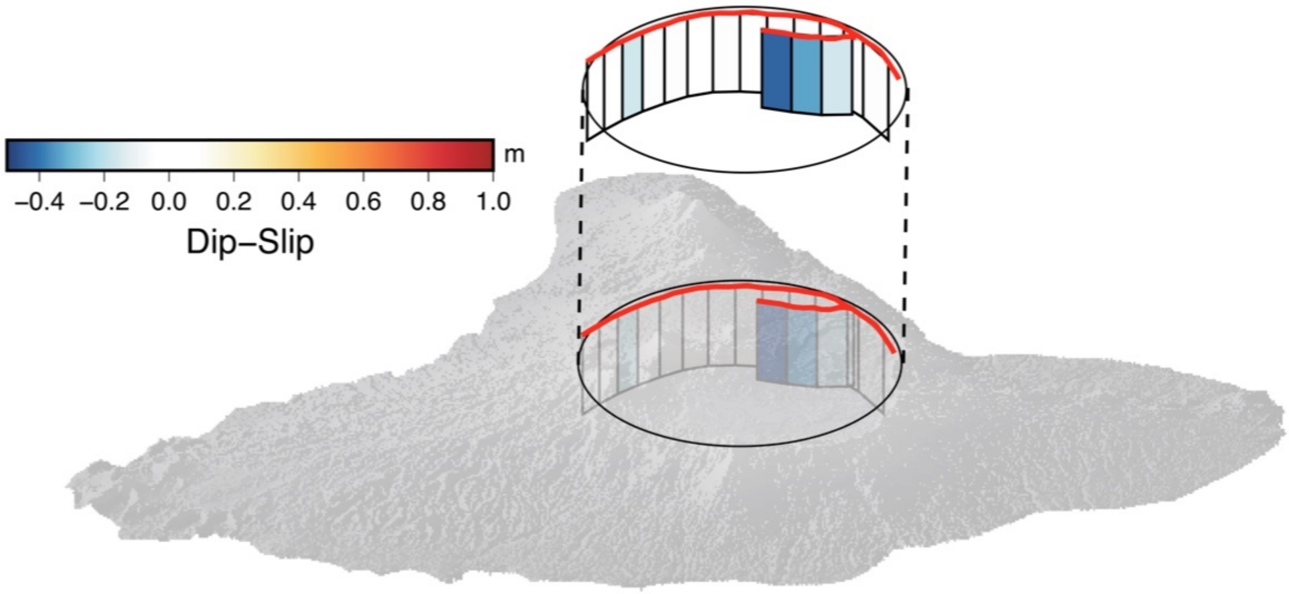

Figure S8. **Caldera ring faulting.** We model the dip-slip component of caldera ring faulting using the distributed opening constrained least squares inversion described in the supplementary text. The strike of these faults was obtained by mapping fringe discontinuities in the 24 Nov – 22 Dec 2018 ALOS-2 ascending interferogram. Vertical fault patches extend to 2 km depth and have a length of 1.04 km along-strike for the outer caldera fault, and 1.17 km along-strike for the inner caldera fault. The model finds a maximum dip-slip (normal faulting) of -0.4 m along the inner caldera fault in the NE portion of the caldera.

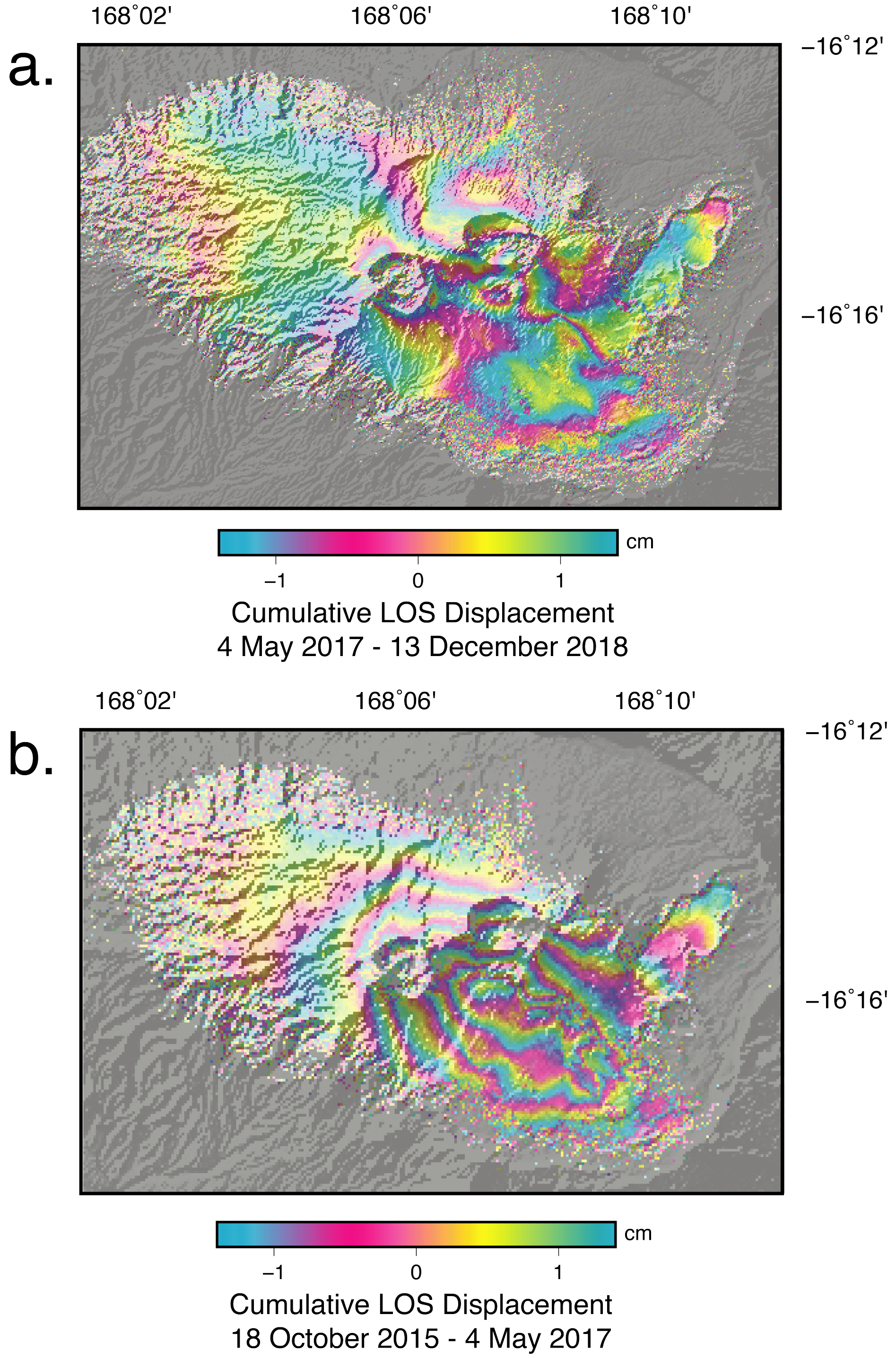

Figure S9. Absence of pre-eruptive uplift. Top panel: A temporal series analysis, calculated using Sentinel-1 interferograms and NSBAS (Doin, et al. 2011), measures very little (if any) pre-eruptive deformation (at most 3 cm uplift centered to the south of Marum). Bottom panel: In the years prior to the 2018 event, subsidence within the caldera dominated during the time period 2015–2017, following the 2015 February eruption, at a rate of approximately -1 cm/month, elongated in the direction of the rift zone.

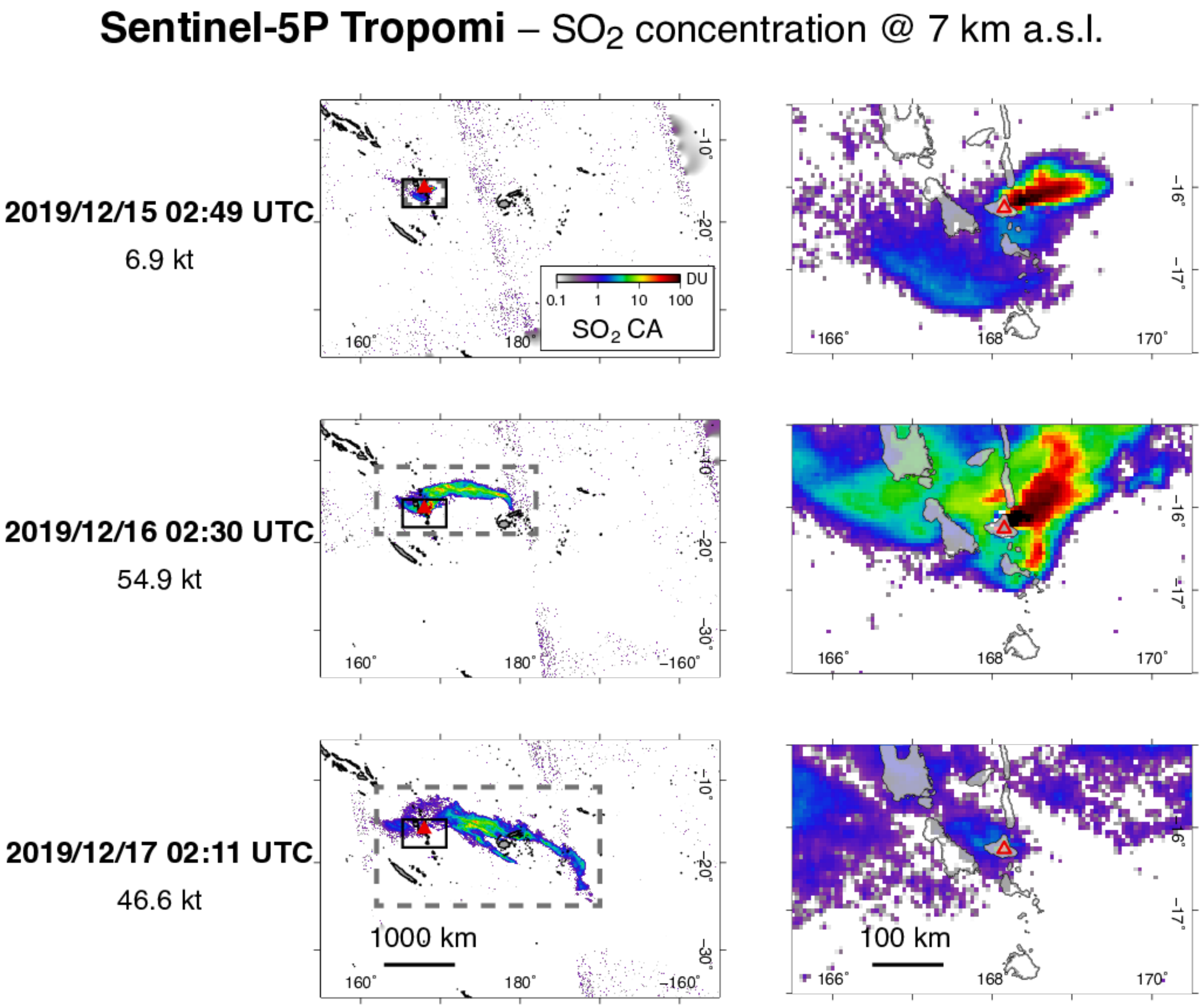

Figure S10. **SO_2 emissions from Sentinel-5P TROPOMI** Daily SO_2 total vertical column density from Sentinel-5P TROPOMI, processed at Level 2. Left: broad-scale view over SW Pacific. Right: detailed view over Ambrym. SO_2 column amount (CA) is estimated assuming a SO_2 plume at 7 km altitude w.r.t. the sea level. Plume altitude is approximately constrained by HYSPLIT simulations of plume dispersal. DU: Dobson Unit. Total burden of SO_2 is estimated by computing a surface integral in the area outlined by the grey dashed rectangles.

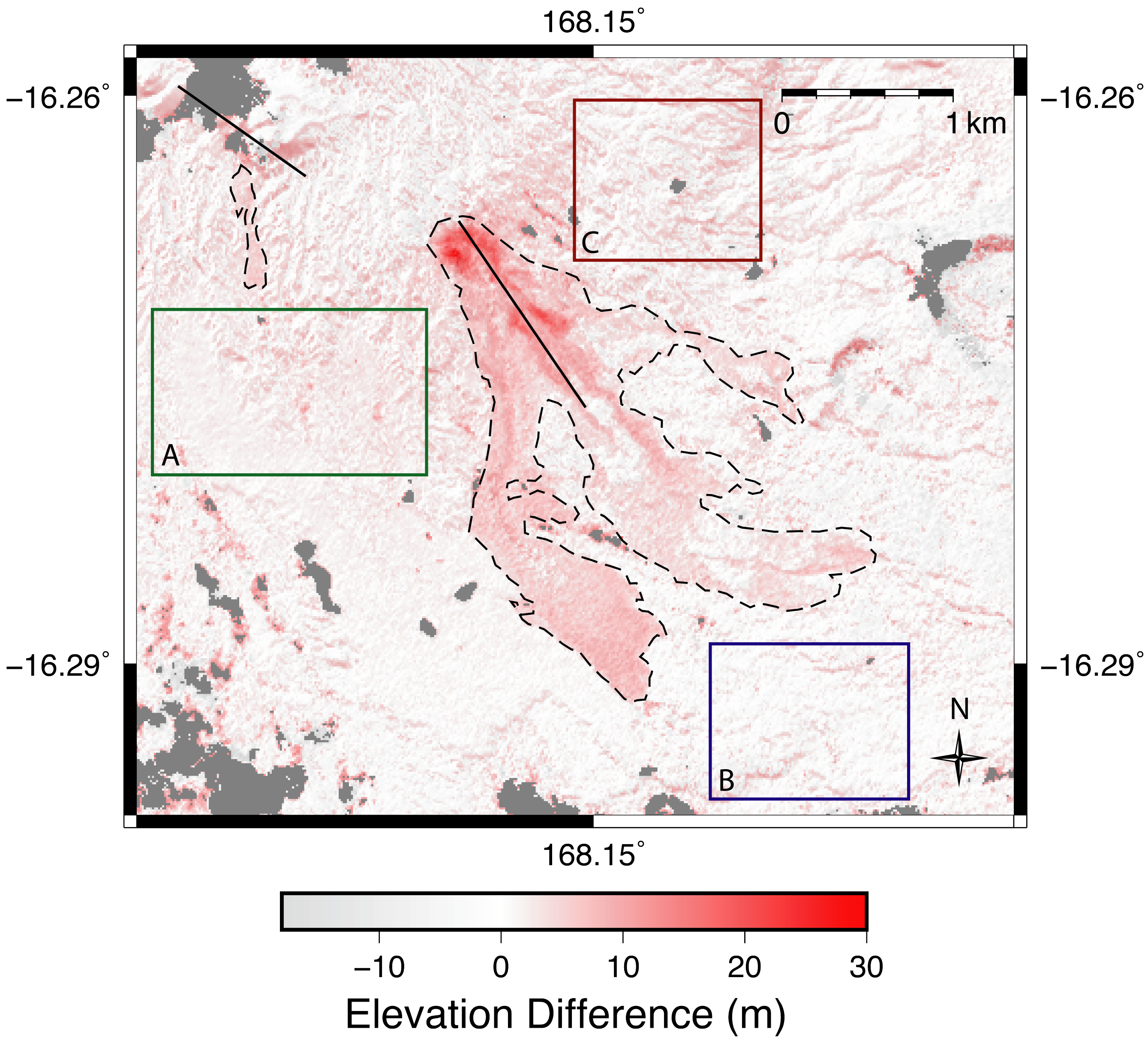

Figure S11. Difference between DEM's pre- and post- February 2015. A DEM difference that measures the extent of the lava flows from the February 2015 eruption[8]. The post-eruption DEM is calculated using Micmac software (https://micmac.ensg.eu)[9] derived from Pleiades optical images. By measuring the lava flow thickness from historical eruptions, we can estimate the volume of lava emitted during the 2018 eruption. The total volume from the 2015 lava flow is calculated to be 12.41×10^6 m^3, with an average lava flow thickness of ~5.17 m. In control areas A, B, and C, where no lava was emplaced, the mean elevation of the DEM difference is 1.11 m, −0.341 m, and 0.239 m, with standard deviations of 1.4 m, 1.64 m, and 1.87 m, respectively. Pre-eruption DEM ©DLR 2017, post-erupton DEM ©CNES 2018, Distribution AIRBUS DS. Map was generated with GMT version 5.4.3 (http://gmt.soest.hawaii.edu) and edited in Adobe Illustrator version 16.0.4 (https://www.adobe.com/products/illustrator.html)

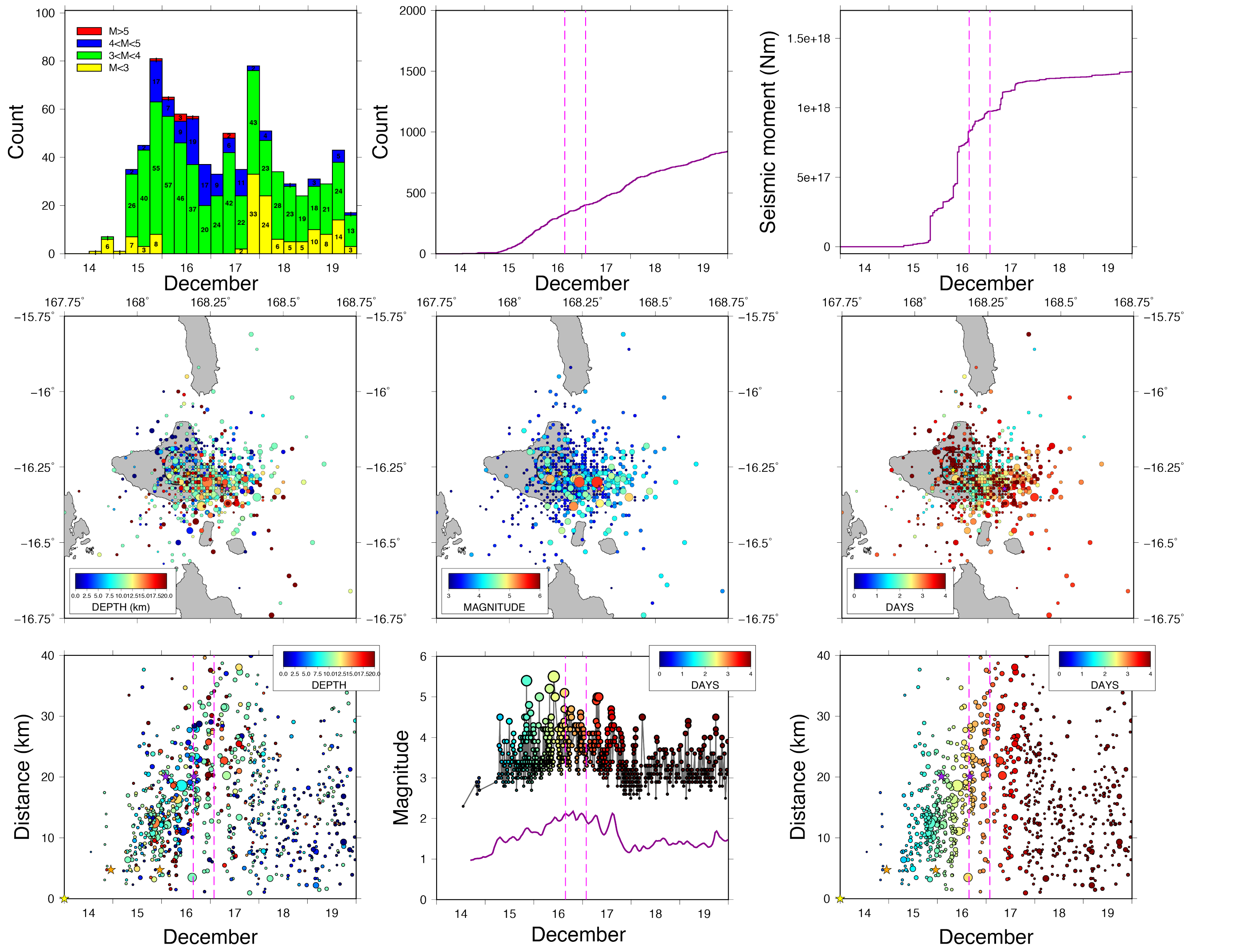

Figure S12. Seismicity during eruption. Details of seismicity reported by Vanuatu Meteorology and Geohazard Department (VMGD). Time span covered by these plots is from 14 December 2018 00:00 UTC to 19 December 2018 23:59 UTC. Only earthquakes with M>2 and depth<60 km are shown. Top left: earthquake count by magnitude. Bin size is 3 hours. Top center: cumulative earthquake count. Dashed pink lines correspong to occurrence of two earthquakes with a P-vertical CLVD mechanism, associated with caldera collapse (Fig. 2b). Top right: cumulative seismic moment release. Middle left: seismicity map with earthquakes colored by depth. Middle center: seismicity map with earthquakes colored by magnitude. Middle right: seismicity map with earthquakes colored by elapsed time from a reference time of 14 December 2018 00:00 UTC. Bottom left: evolution of seismicity as a function of distance from Marum, with earthquakes colored by depth. Bottom center: evolution of seismicity as a function of magnitude, with earthquakes colored by date. Bottom right: evolution of seismicity as a function of distance from Marum, with earthquakes colored by date.

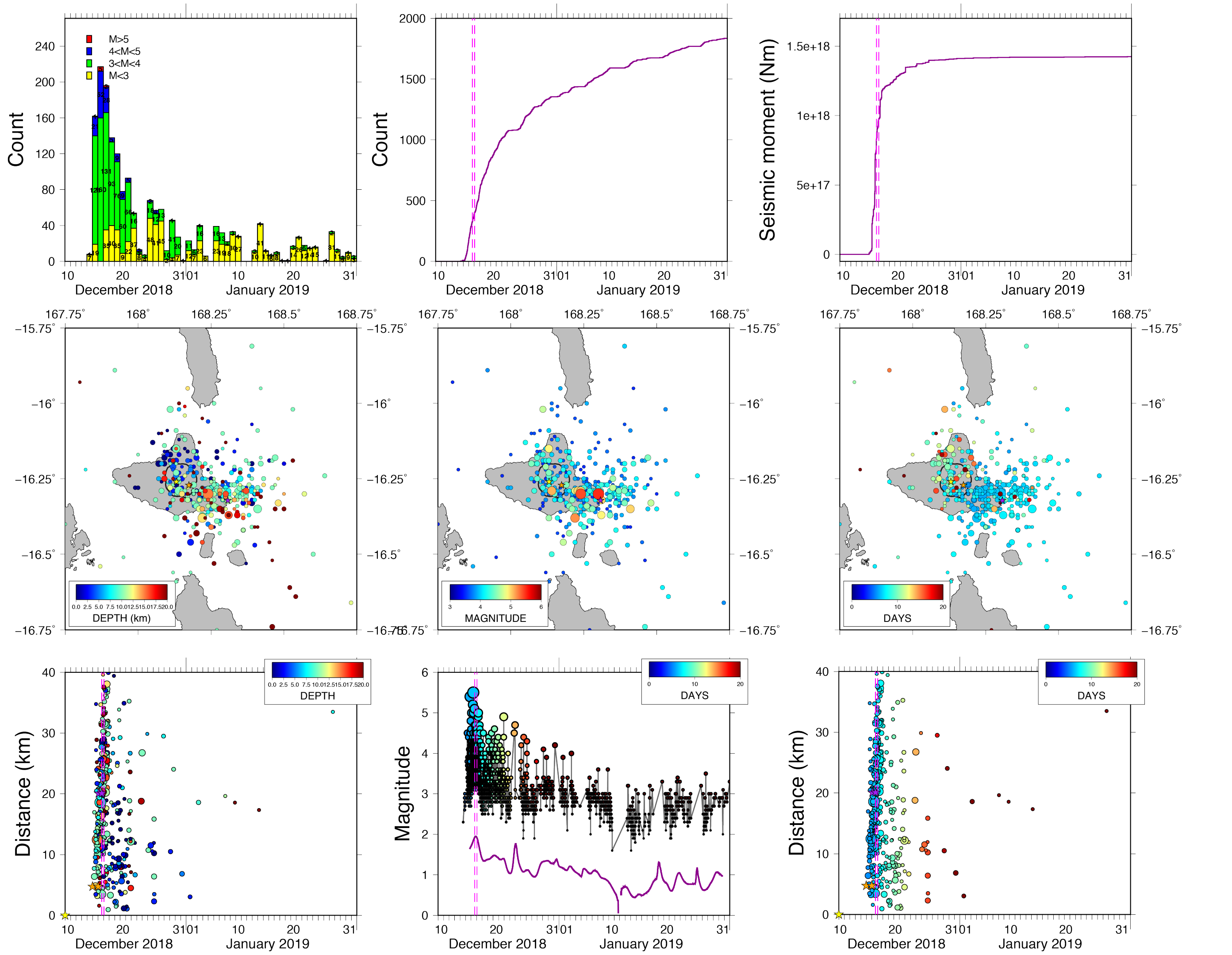

Figure S13. **Seismicity during eruption and after event.** Same as Fig. S12, but for the time interval from 10 December 2018 00:00 UTC to 31 January 2019 23:59 UTC, for earthquakes with M>3.5.

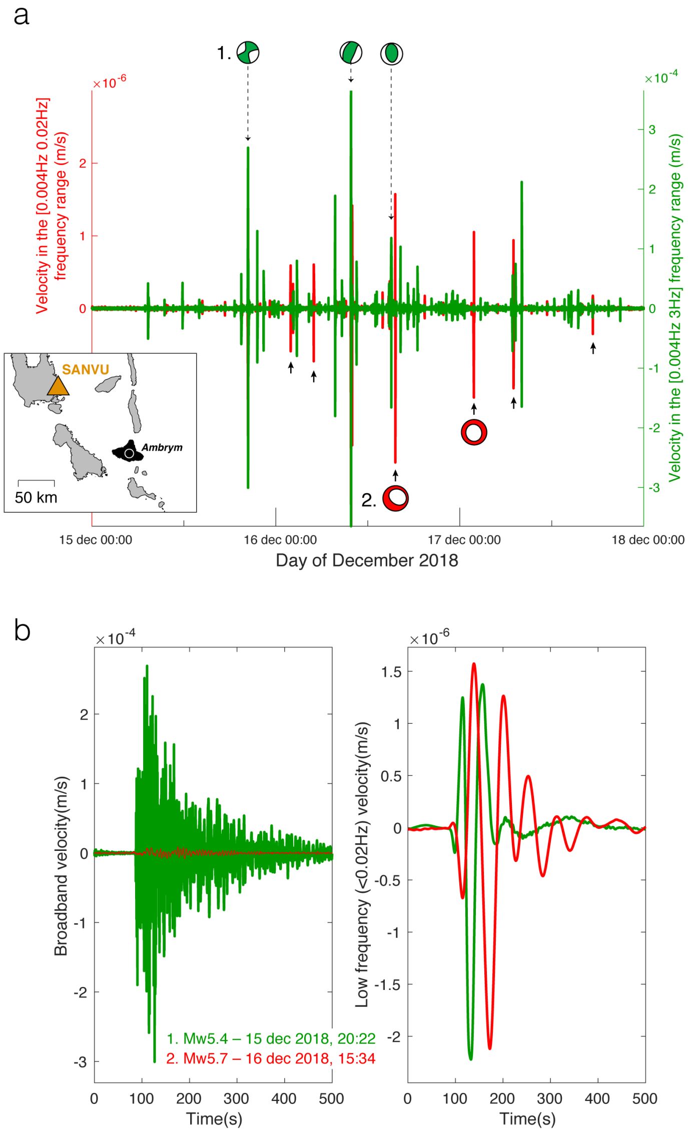

Figure S14. **Broadband and low frequency seismograms. a** Comparison between broadband (green) and low frequency (red) velocity seismograms at Geoscope station SANVU[10]. The raw record has first been corrected for instrument response and high-pass filtered at 0.004 Hz (250 seconds). The broadband and low frequency seismograms have then been obtained after a lowpass filter at 3Hz and 0.02Hz (0.3 seconds and 50 seconds), respectively. Green and red focal mechanisms of the largest events are from USGS and Global CMT, respectively, as in Fig. 2. Note the different vertical scales for the broadband and filtered seismograms. Peaks in the broadband (green) seismograms indicate events with a high frequency content (volcano-tectonic events, VT) associated with the Ambrym seismic crisis. Black arrows indicate events with a ratio of low-frequency-to-high-frequency exceeding significantly the average inferred from VT (red spikes), indicating a low frequency content (long-period events, LP). Station location is shown in inset. **b** Same as a., but keeping an identical vertical scale. Here, two events with contrasting low-frequency-to-high-frequency ratios are compared. The first event (1) is the main VT event of 15 December 2018 (Mw5.4, 15 December 2018, 20:22). The second event (2) is the first LP event of 16 December 2018 with a P-vertical CLVD mechanism reported by Global CMT (Mw5.7 16 December 2018, 15:34). The LP event produces a much smaller signature in the broadband seismogram (left). In contrast, after low-pass filtering, both events show a similar amplitude (right). In addition, the clear differential signal duration between both events is a direct evidence of a long source process (at least several tens of seconds) for the Mw5.7 LP event.

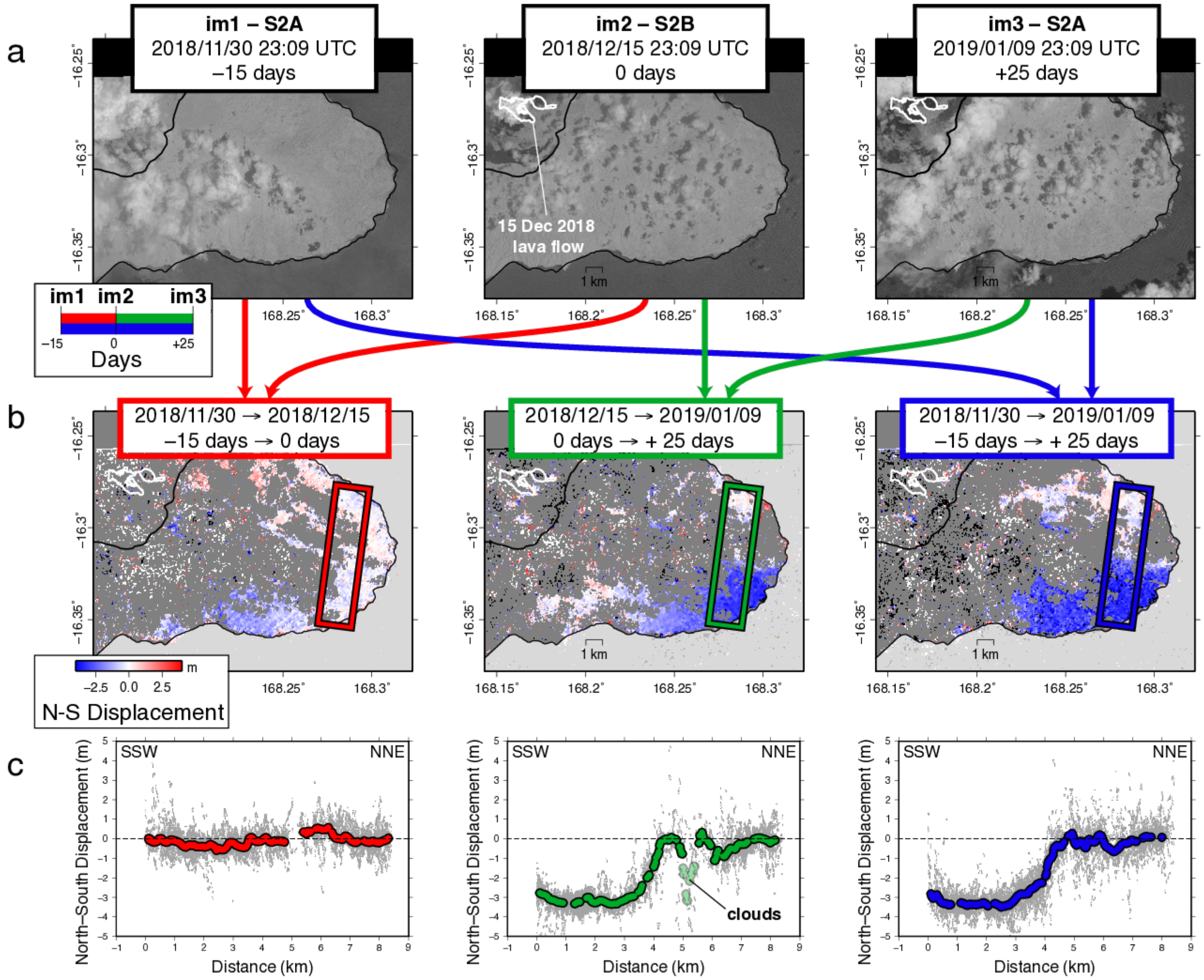

Figure S15. **Sentinel-2 optical image correlation.** Sub-pixel correlation of Sentinel-2 optical images covering the eastern rift zone of Ambrym with mild cloud cover. Images are acquired on (a) 30 November 2018, (b) 15 December 2018, 23:09 UTC, and (c) 9 January 2019. Upper row shows a preview of images for band 8 (near-infrared, 767–908 μm) which is characterized by a high reflectivity in vegetated area. Image resolution is 10 meters. Central row is the result of pair-wise sub-pixel correlation, processed with MicMac software[9] (`https://micmac.ensg.eu`). Lower row shows swath profiles in the boxes indicated by the rectangles in the central row. Grey points show raw results in a box spanning +/-1 km from a reference profile. The location of the box is shown in b. The colored curves are computed by applying a 0.2 km-wide median filter to the raw data. Negative (blue): motion toward the south. Positive (red): motion toward the north.

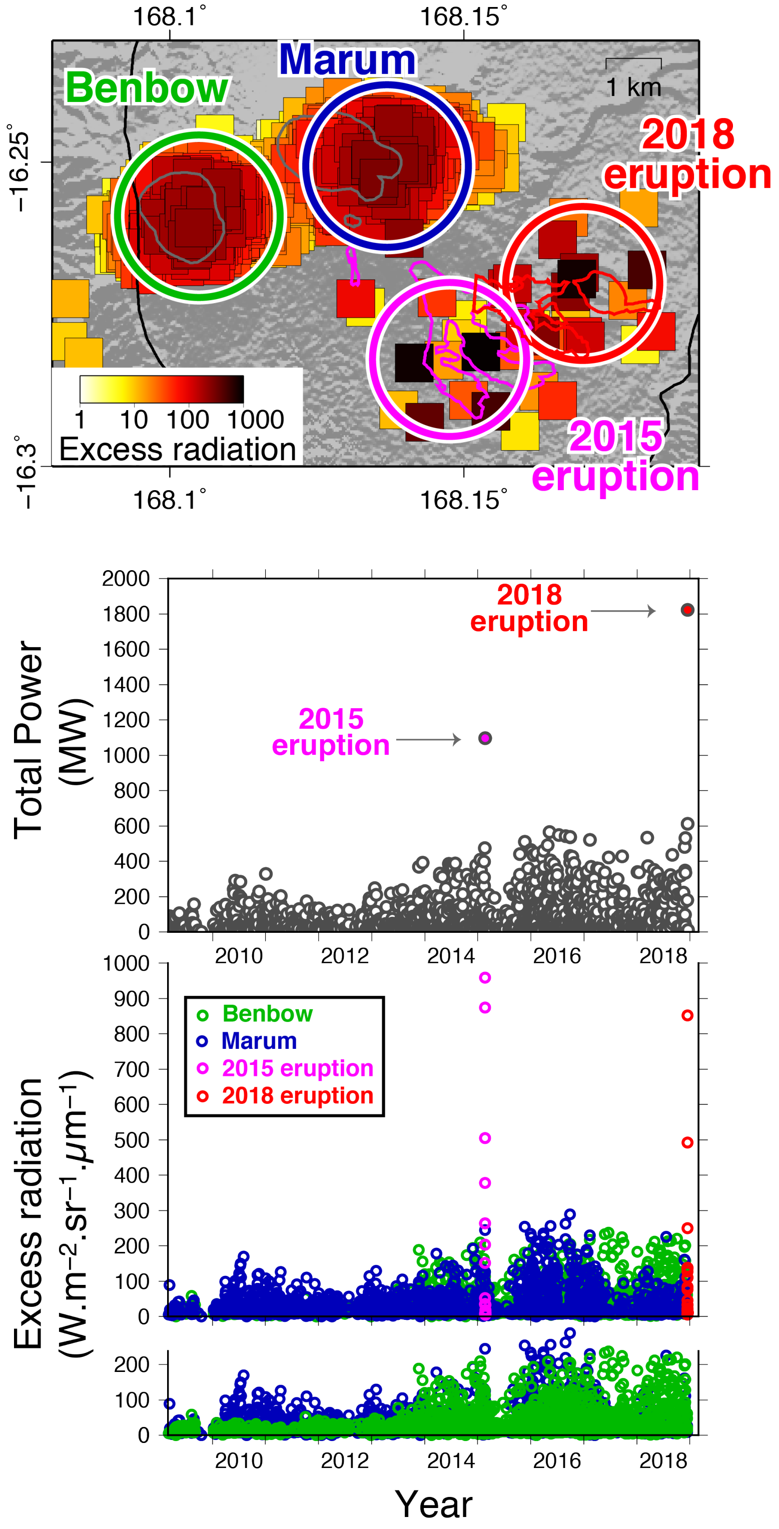

Figure S16. **10-year MODIS thermal activity.** Time-series of excess thermal radiation and total radiated power from 2009 to 2019, derived from MODIS (Wright et al., 2016). Upper panel shows the spatial distribution of thermal anomalies. Middle graph shows the total power over the whole Ambrym caldera as a function of time. Lower graph shows excess radiation as a function of time, colored according to the location of the radiation (green: Benbow; blue: Marum; pink: 2015 eruption; red: 2018 eruption). Circles in the upper panel indicate the spatial regions corresponding to each of the 4 locations. Processed data is from the MODVOLC service (`http://modis.higp.hawaii.edu/algorithm.html`)[6].

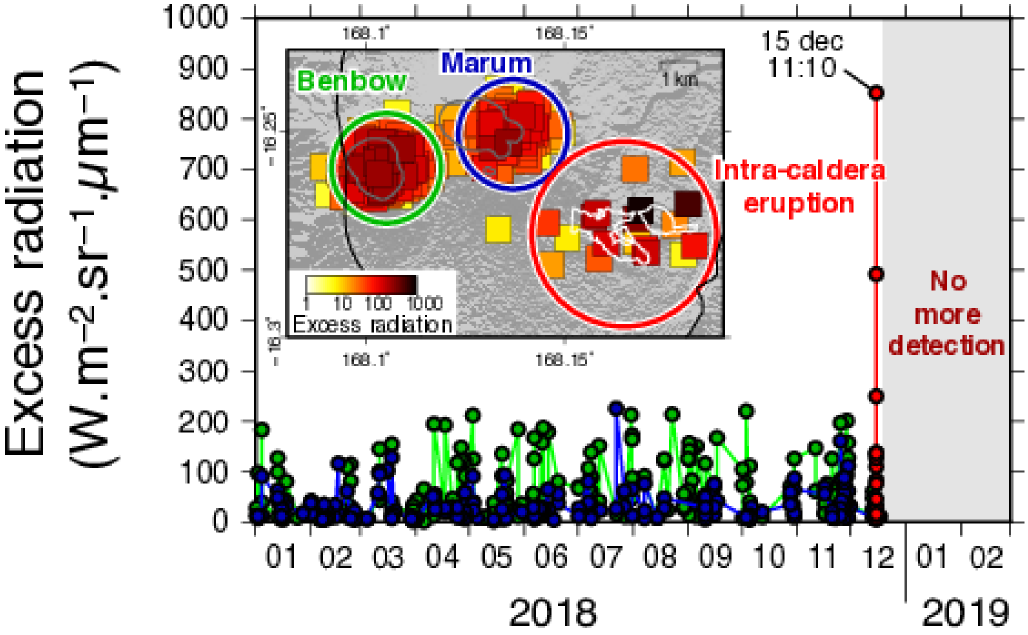

Figure S17. 1-year MODIS thermal activity. Same as Fig. S16a for the period between 1 January 2018 and 28 February 2019. Note that no anomaly was detected after the last detection of 17 December 2018 14:05 UTC.

Table S1. SAR data used in inversions

Inversion	Master date (UTC)	Slave date (UTC)	Sensor	Mode	Geometry/Track	Data type
Intra-caldera dike	2018/11/03 00:24	2018/12/15 00:24	ALOS-2	Wideswath (WD1)	Descending T203	InSAR
Rift zone intrusion and caldera subsidence	2018/11/24 13:14	2018/12/22 13:14	ALOS-2	Stripmap (SM3)	Ascending T101	InSAR
	2018/12/15 00:24	2019/01/26 00:24	ALOS-2	Wideswath (WD1)	Descending T203	InSAR
	2018/12/14 06:10	2018/12/18 06:10	CSK	Stripmap (H4-04)	Descending	Azimuth/range pixel offsets
Post-intrusion caldera subsidence	2018/12/22 13:14	2019/02/16 13:14	ALOS-2	Stripmap (SM3)	Ascending T101	InSAR
	2018/12/18 06:10	2019/01/15 06:10	CSK	Stripmap (H4-04)	Descending	Azimuth/range pixel offsets

Table S2. **First-order geometries derived from non-linear inversion.**

Inversion	Source type	Center position (lat/lon)	Strike	Dip	Depth (km)	Length (km)	Width (km)	Opening (m) / Volume (km^3)
Intra-caldera dike	Okada	168.165°E −16.267°N	88°	38°	0.4	3.25	3.46	1.96/0.022
Rift zone intrusion and caldera subsidence	Okada	168.165°E −16.267°N	109°	72°	0.0†	27.36	6.94	4.0/0.76
	Mogi	168.136°E −16.253°N	N/A	N/A	4.5	N/A	N/A	N/A/−0.231
Post-intrusion caldera subsidence	Okada	168.146°E −16.255°N	−41°	0.0†	4.1	7.74	6.14	−1.30/0.062

† : fixed.

Table S3. **Sentinel-1 acquisition dates and scalars.**

Master date	Slave date	Scalar
2018/12/19	2018/12/25	0.55
2018/12/19	2018/12/31	0.87
2018/12/19	2019/01/06	1.03
2018/12/19	2019/01/12	1.04
2018/12/19	2019/01/18	1.09
2018/12/25	2018/12/31	0.29
2018/12/25	2019/01/06	0.41
2018/12/25	2019/01/12	0.47
2018/12/25	2019/01/18	0.52
2018/12/31	2019/01/06	0.11
2018/12/31	2019/01/12	0.17
2018/12/31	2019/01/18	0.23
2019/01/06	2019/01/12	0.06
2019/01/06	2019/01/18	0.12
2019/01/12	2019/01/18	0.06
2018/12/22	2019/01/15	1.00‡
2018/12/22	2019/02/16	1.00‡

‡ : reference (fixed).

Table S4. **Average major element composition for lava erupted during the intra-caldera eruption at Lewolembwi**. Given the very low crystal content ($< 5\%$) of the rock samples and the close correspondence between melt inclusions and embayment and matrix glasses composition, these analyses can also be considered representative of the bulk rock composition.

Sample type	SiO_2	TiO_2	Al_2O_3	FeO	MnO	MgO	CaO	Na_2O	K_2O	P_2O_5	Total
Melt inclusions (N=9)	53.30	1.17	16.09	10.80	0.20	3.50	8.77	3.23	2.48	0.45	100.00
Embayments and matrix glasses (N=6)	53.24	1.08	16.32	10.90	0.23	3.62	8.60	3.16	2.43	0.42	100.00

References

1. Grandin, R. *et al.* September 2005 Manda hararo-dabbahu rifting event, Afar (Ethiopia): Constraints provided by geodetic data. *J. Geophys. Res. Solid Earth* **114**, DOI: 10.1029/2008JB005843 (2009).
2. Radiguet, M. *et al.* Spatial and temporal evolution of a long term slow slip event: The 2006 Guerrero Slow Slip Event. *Geophys. J. Int.* **184**, 816–828, DOI: 10.1111/j.1365-246X.2010.04866.x (2011).
3. Marty, J. *et al.* Gins: the cnes/grgs gnss scientific software. In *3rd International Colloquium Scientific and Fundamental Aspects of the Galileo Programme, ESA Proceedings WPP326*, vol. 31, 8–10 (2011).
4. Böhm, J., Niell, A., Tregoning, P. & Schuh, H. Global mapping function (gmf): A new empirical mapping function based on numerical weather model data. *Geophys. Res. Lett.* **33** (2006).
5. Lagler, K., Schindelegger, M., Böhm, J., Krásná, H. & Nilsson, T. Gpt2: Empirical slant delay model for radio space geodetic techniques. *Geophys. research letters* **40**, 1069–1073 (2013).
6. Wright, R., Flynn, L., Garbeil, H., Harris, A. & Pilger, E. Automated volcanic eruption detection using MODIS. *Remote. sensing environment* **82**, 135–155 (2002).
7. Planet Team. Planet Application Program Interface: In Space for Life on Earth (2017). San Fransisco, CA. https://api.planet.com.
8. Coppola, D., Laiolo, M. & Cigolini, C. Fifteen years of thermal activity at Vanuatu's volcanoes (2000 - 2015) revealed by MIROVA. *J. Volcanol. Geotherm. Res.* **322**, 6–19, DOI: 10.1016/j.jvolgeores.2015.11.005 (2016).
9. Rosu, A. M., Pierrot-Deseilligny, M., Delorme, A., Binet, R. & Klinger, Y. Measurement of ground displacement from optical satellite image correlation using the free open-source software MicMac. *ISPRS J. Photogramm. Remote. Sens.* **100**, 48–59, DOI: 10.1016/j.isprsjprs.2014.03.002 (2015).
10. Institut de Physique du Globe de Paris and Ecole et Observatoire des Sciences de la Terre de Strasbourg (EOST). GEOSCOPE - French Global Network of broadband seismic stations, DOI: 10.18715/GEOSCOPE.G (1982).
